# Fetoscopic laser versus amnioreduction, septostomy, and expected management for the treatment of twin-twin transfusion syndrome (TTTS): an economic evaluation analysis in Iran

**DOI:** 10.1186/s12962-024-00551-2

**Published:** 2024-05-09

**Authors:** Zhila Najafpour, Kamran Shayanfard, Negar Aghighi, Najmieh Saadati

**Affiliations:** 1 Department of Health Care Management, School of Public Health, Ahvaz Jundishapur University of Medical Sciences, Ahvaz, Iran; 2 Physics and Materials Science Research Unit, University of Luxembourg, Luxembourg, Luxembourg; 3 Vice Chancellor of Treatment, Health Services Management, Hamedan University of Medical Sciences, Hamedan, Iran; 4 Department of Obstetrics and Gynecology, School of Medicine, Ahvaz Jundishapur University of Medical Sciences, Ahvaz, Iran

**Keywords:** Amnioreduction, Cost-effectiveness analysis, Expected management, Fetoscopic laser, Septostomy, Twin-twin transfusion syndrome

## Abstract

**Background:**

Twin-twin transfusion syndrome (TTTS) affects 10–15% of monochorionic twin pregnancies. Without treatment, their mortality rates would be considerable. There are differences in survival rate between different therapeutic modalities. This study aims to compare the cost-effectiveness of Fetoscopic laser versus amnioreduction, septostomy, and expected management in the treatment of twin-to-twin transfusion syndrome (TTTS).

**Methods:**

This is a cost-effectiveness analysis of the treatment strategies in patients with TTTS. A decision tree model was used to estimate the clinical and economic outcomes with a pregnancy period time horizon. Medical direct costs were extracted in a quantitative study, and survival rates were determined as effectiveness measures based on a review. A probabilistic sensitivity analysis was used to measure the effects of uncertainty in the model parameters. The TreeAge, Excel and R software were used for analyzing data.

**Results:**

In the first phase, 75 studies were included in the review. Based on the meta-analysis, a total of 7183 women treated with Fetoscopic laser, the perinatal survival of at least one twin-based pregnancy was 69%. In the second phase, the results showed that expected management and amnioreduction have the lowest (791.6$) and highest cost (2020.8$), respectively. Based on the decision model analysis, expected management had the lowest cost ($791.67) and the highest rate in at least one survival (89%), it was used only in early stages of TTTS. Fetoscopic laser surgery, with the mean cost 871.46$ and an overall survival rate of 0.69 considered the most cost-effectiveness strategy in other stages of TTTS.

**Conclusion:**

Our model found Fetoscopic laser surgery in all stages of TTTS to be the most cost-effective therapy for patients with TTTS. Fetoscopic laser surgery thus should be considered a reasonable treatment option for TTTS.

**Supplementary Information:**

The online version contains supplementary material available at 10.1186/s12962-024-00551-2.

## Introduction

Twin-twin transfusion syndrome (TTTS) affects 10–15% of monochorionic twin pregnancies. This syndrome results from an unbalanced blood flow exchange through vascular communications in the monochorionic placenta. This situation results in a volume depleted in donor twin and a volume overload in recipient twin. Without treatment, mortality rate exceed 90%, and approximately 15–50% of survivors may have a long-term disability [1]. The poor outcome of untreated TTTS caused to emerge of several therapeutic modalities: amnioreduction, endoscopic laser photocoagulation of vascular anastomoses, septostomy, expected management, and selective feticide [2].

During the early 1990s, with advances in ultrasound technology and the development of prenatal therapies, serial amnioreduction was the only treatment option for TTTS [3]. Based on a review, survival rates following serial amnioreduction were 67% [4], with a risk of neurological damage 30% [5]. It is noteworthy that amnioreduction ameliorated the disease by temporarily reducing pressure on the chorionic plate, improving placental blood flow. Since the early 2000s, after the introduction of laser surgery, addressing the underlying cause of TTTS, making it the gold standard for treating TTTS between 16 and 28 weeks of gestation [3]. Based on literature, laser surgery resulted in survival rates for at least one twin ranging from 65 to 93% and dual survival rates ranging from 18–62% [6, 7], with a 10% neurological handicap rate [8]. Another therapeutic option is septostomy which designates intentionally perforating the membrane separating the twins to allow equilibration of the amniotic fluid volume in TTTS. Survival rate in septostomy is reported 83%, but evidence for neurological outcome was rare. It needs to mention that septostomy may happen during the operation by the laser fiber tip or trocar tip for TTTS. The reported incidence of septostomy after laser therapy ranges from 1.6 to 25.0%. It is associated with several risks, such as early delivery, intrauterine fetal demise (IUFD), and cerebral injury [9, 10]. Expectant management is another modality for patients in the early stage of TTTS. Although due to the concern of progression to higher stages, some studies recommend treating patients in stage 1 TTTS with laser. Based on a recent trial, there was no difference in intact survival between laser surgery and expectant management. They reported a progression to higher stages in 60% of the cases that managed expectantly [11, 12].

From an economic evaluation perspective, the different treatment strategies have varying costs and clinical value. Most treatment strategies are expensive and need a large number of resources. Therefore, it needs to compare costs and effects of the technologies to determine the most cost-effective therapeutic modalities. Based on our knowledge, there is only two cost-effectiveness studies in this field that were conducted based on simulation in patients with TTTS [13, 14]. Then we aimed to perform a cost-effectiveness analysis on the four treatment modalities (Fetoscopic laser, amnioreduction, septostomy, and expected management) in patients with TTTS to assess each treatment modality’s value, costs, and cost-effectiveness ratio.

## Materials and methods

We conducted an economic evaluation study in three phases: a systematic review and meta-analysis for comparing perinatal survival (clinical data), a descriptive cross-sectional study to trace the direct medical costs of different treatment strategies, and a cost-effectiveness analysis using a decision-analytic model of the four treatment modalities in patients with TTTS.

### Systematic review

Articles published in English and indexed in Medline, Cochrane, PubMed, Scopus, web of science, and Google Scholar were searched. Studies published before 2000 were also excluded, since advances in prenatal imaging techniques and improvements in the diagnosis and treatment of TTTS make them less relevant. Two authors (ZN and NA) performed the search independently. Our search was performed with combinations of the following keywords: survival, perinatal survival, twin-to-twin transfusion syndrome, TTTS, twin-twin transfusion syndrome, pregnancy reduction, monochorionic pregnancy, monochorionic twin, Fetofetal transfusion, laser, laser therapy, laser ablation, selective laser, sequential laser, laser coagulation, Fetoscopy, photocoagulation, endoscopic laser, amnioreduction (see the complete search strategy in the supplementary 1).

### Inclusion and exclusion criteria

Randomized trials, observational and comparative studies, prospective and retrospective case series were considered eligible for inclusion. Reasons for exclusion were studies with insufficient data, letters, conference abstracts, and case reports. Meanwhile, non-English articles were excluded.

### Study selection and data extraction

Two reviewers independently screened all references (ZN and NA). Agreement regarding potential relevance was reached by consensus. Disagreement on the eligibility of a study was resolved by discussion with third researchers (KS). They also extracted relevant data based on pre-defined measures. Our extracted data were the survival rate of one twin and the survival rate of at least one twin. Other parameters were complications, such as preterm premature rupture of membranes (PPROM), twin anemia-polycythemia sequence (TAPS), placental abruption, cerebral lesions, and Cerebral palsy. We did not consider different types of laser techniques and also extracted the occurrence of mortality and morbidity in twins until the perinatal period.

### Data synthesis and analysis

Survival rates of different modalities were calculated, and the 95% confidence intervals (CIs) were estimated using a binomial distribution. Random-effect meta-analyses of proportions were used to combine data. For the analysis, the denominator was represented by the number of twins per modality for the computation of survivors and morbidity. Our results have been reported in two categories included RCT and observational studies for each modality. Between-study heterogeneity was explored using the I2 statistic, which represents the percentage of between-study variation that is due to heterogeneity rather than chance. All analyses were performed using R Software.

### Cost measurements

Cost of care in patients with TTTS were identified from the health system’s perspective with a bottom-up approach. Cost components included only direct medical costs (DMC) estimated from the patient’s medical records and expert opinions based on the prices of 2022. Components of DMC included the cost of hospitalization, surgery, physician visits, medications, consuming materials, services of nursing, and all diagnostic medical services for each modality. DMC was extracted based on all related costs of patients with TTTS who had been referred to the Shariati hospital as a single referral hospital for patients with TTTS in Iran. All DMC included all clinical costs, the average use percentage of each service, and the price of each service based on the tariffs of the year 2022 was entered in a form designed by experts after checking patients’ medical records, patients’ bills, and expert opinions.

### Cost-effectiveness analysis

Regarding the obtained costs and effectiveness measures extracted, we assessed the cost-effectiveness of each treatment modality. The time frame of our analysis was the pregnancy period (or 40 weeks). The discount rate was not used because the study horizon was less than one year. We measured the cost-effectiveness ratio with at least one survival and death rate. Then we compared four treatment modalities.

### Decision tree model

**Fig. 1:**
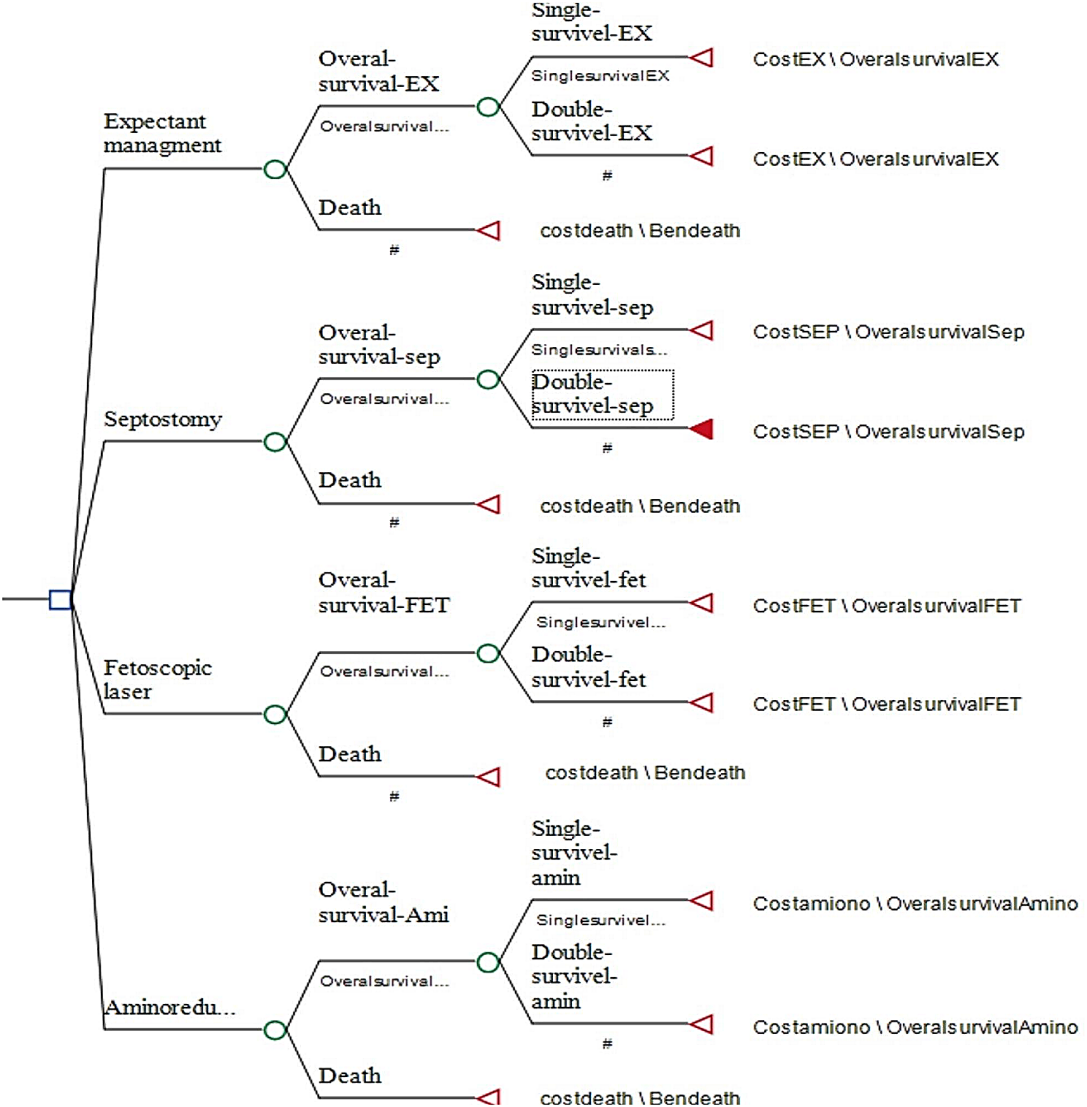
Decision tree model diagram

The decision tree model was used to estimate the cost and effectiveness (survival rate) in patients with TTTS. Our modalities to treat the patients with TTTS were divided into four subgroups: (1) Fetoscopic laser; (2) AD; (3) septostomy; (4) expected management. Costs and outcomes for each branch were extracted from the previous phase. Meanwhile, we considered utility 1 for survival and 0 for death. The model was designed in TreeAge software in 2011. The incremental cost-effectiveness ratio (ICER) was calculated based on extracted data. ICER was estimated using the following formula. Figure 1 presents the model used in this study.

$$ICER=\frac{\text{C}\text{o}\text{s}\text{t}\, \text{n}\text{e}\text{w}\, \text{t}\text{r}\text{e}\text{a}\text{t}\text{m}\text{e}\text{n}\text{t}-\text{C}\text{o}\text{s}\text{t}\, \text{o}\text{l}\text{d}\, \text{t}\text{r}\text{e}\text{a}\text{t}\text{m}\text{e}\text{n}\text{t}}{\text{E}\text{f}\text{f}\text{e}\text{c}\text{t}\text{i}\text{v}\text{e}\text{n}\text{e}\text{s}\text{s}\, \text{n}\text{e}\text{w}\, \text{t}\text{r}\text{e}\text{a}\text{t}\text{m}\text{e}\text{n}\text{t}-\text{E}\text{f}\text{f}\text{e}\text{c}\text{t}\text{i}\text{v}\text{e}\text{n}\text{e}\text{s}\text{s}\, \text{o}\text{l}\text{d}\, \text{t}\text{r}\text{e}\text{a}\text{t}\text{m}\text{e}\text{n}\text{t}}$$

### Sensitivity analysis

In this study, a tornado diagram was plotted to evaluate the effects of critical parameters of the model results. All variables of clinical values and cost were considered as a distribution. It is also noteworthy that in the present study, beta (β) distributions were used to determine the probability distribution of clinical values ​​and gamma distribution to determine the distribution of cost values. The range was calculated based on the variance and the standard deviation of extracted data in the previous phases.

## Results

**Fig. 2:**
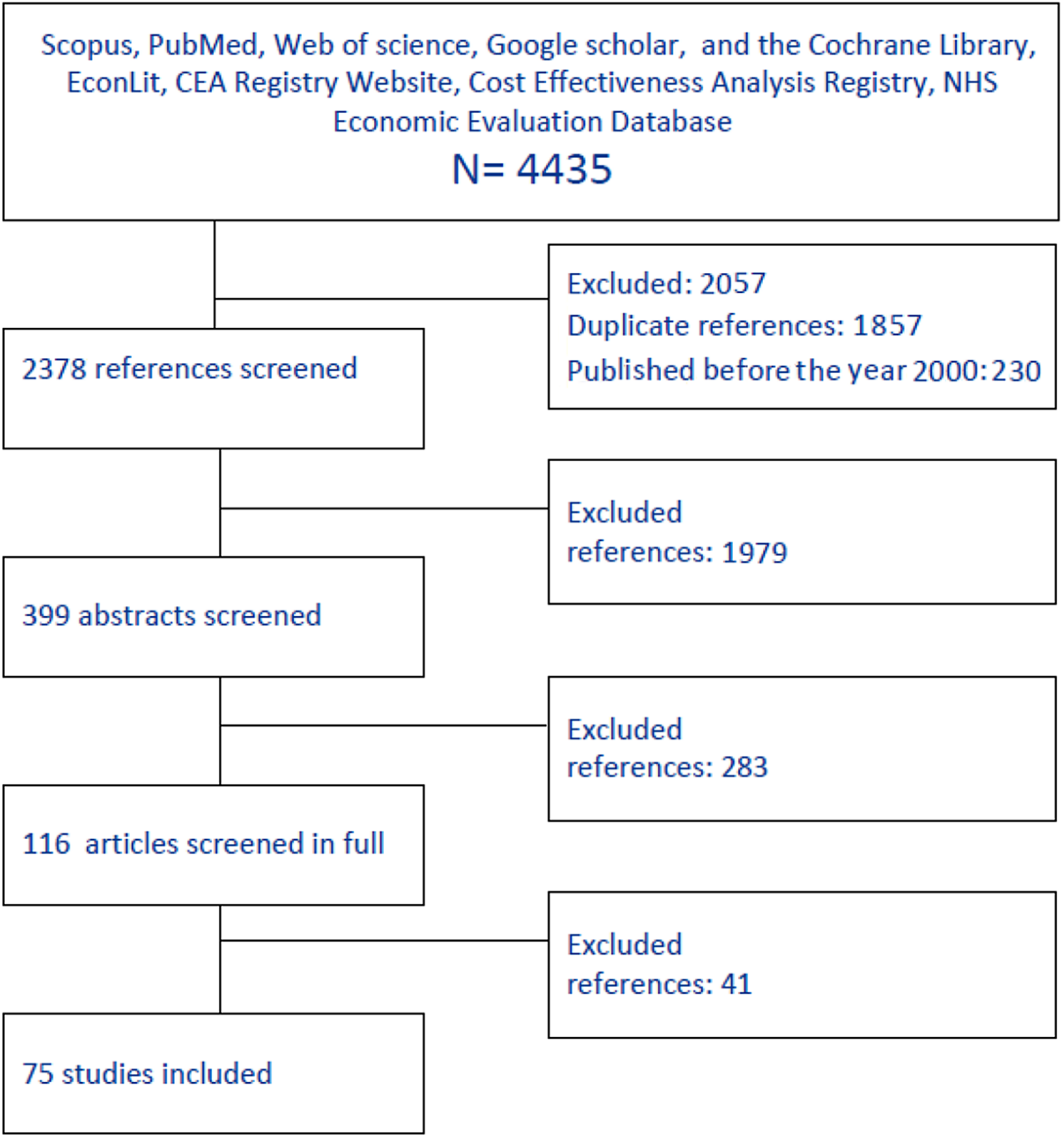
PRISMA flow diagram

4435 articles were identified after a systematic search in the electronic databases. After removing the duplicates (2087), the title and abstract reviewed and finally 75 full texts were included in the study that 49 articles of them reported at least on survivals of modalities. Overall, 39 used Fetoscopic laser, six used amnioreduction, two used septostomy, and two used expected management in patients with TTTS. Several articles considered the different combinations of modalities, these articles included separately in each modality analysis. Additionally, 34 included articles reported complications after each modality (see Fig. 2 and Supplementary 2).

**Fig. 3:**
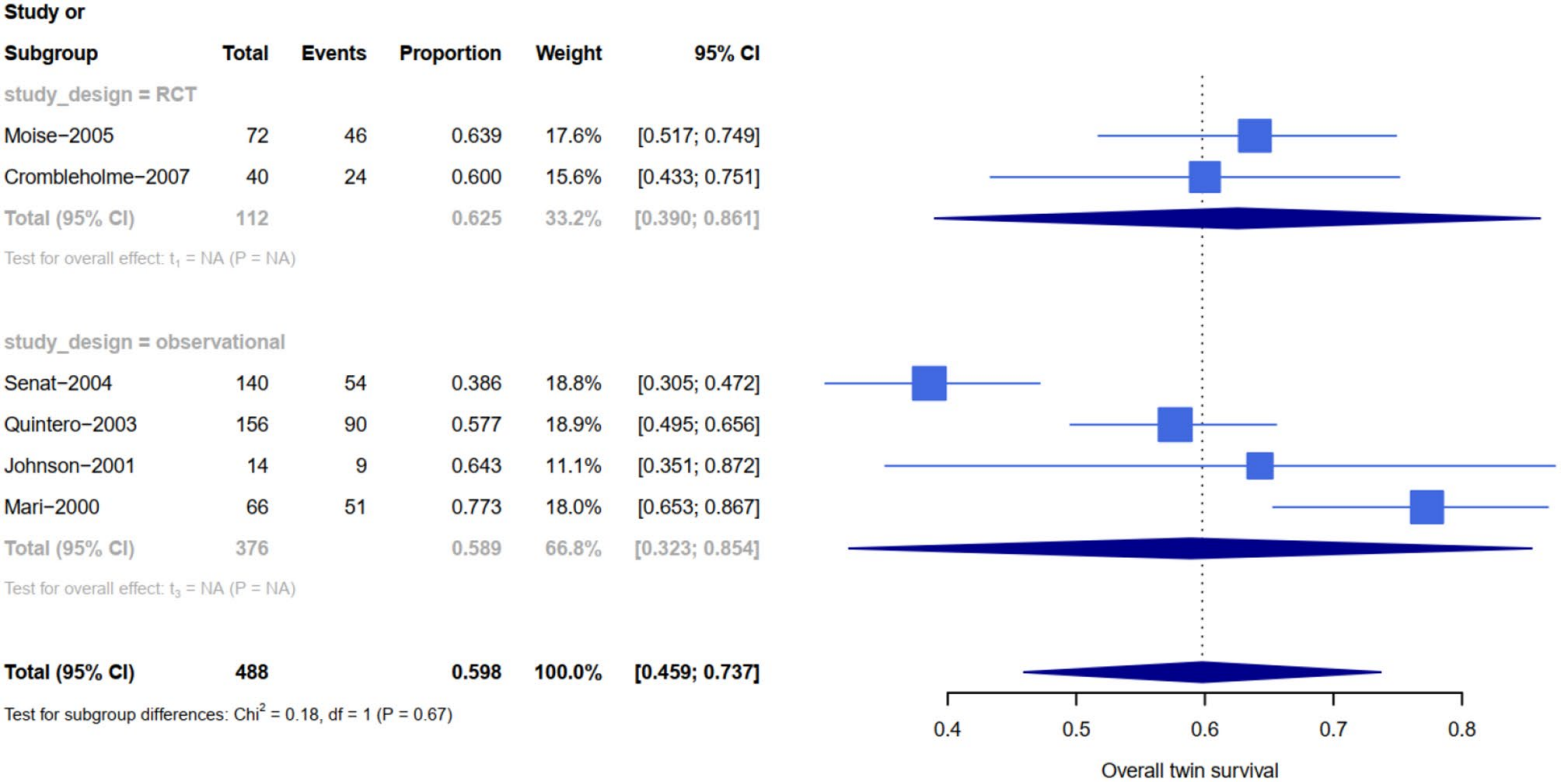
Forest plot showing at least on survival rates subdivided by type of studies of amnioreduction for TTTS

**Fig. 4:**
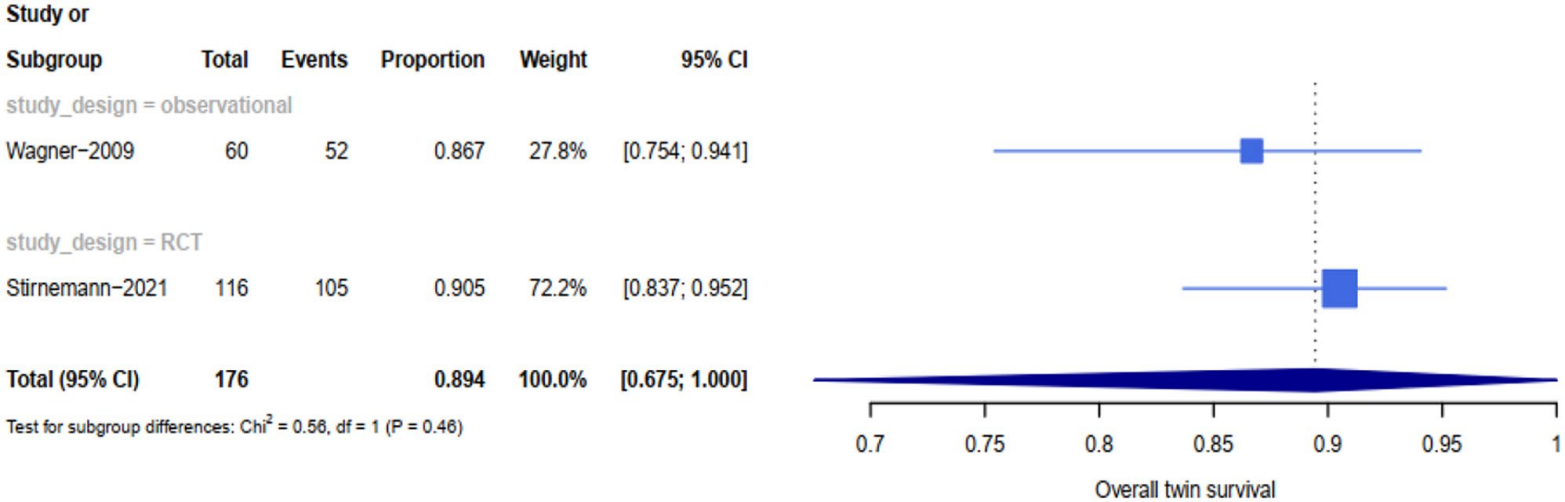
Forest plot showing at least on survival rates subdivided by type of studies of expectant management for TTTS

**Fig. 5:**
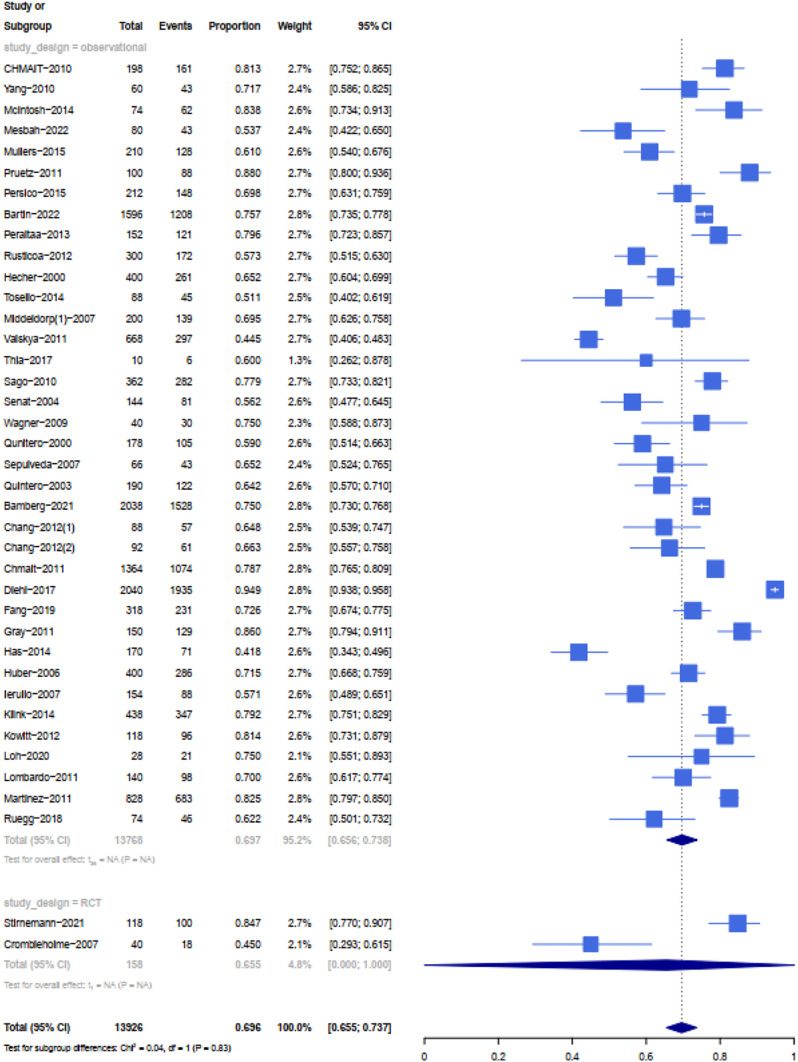
Forest plot showing at least on survival rates subdivided by type of studies of laser surgery for TTTS

**Fig. 6:**
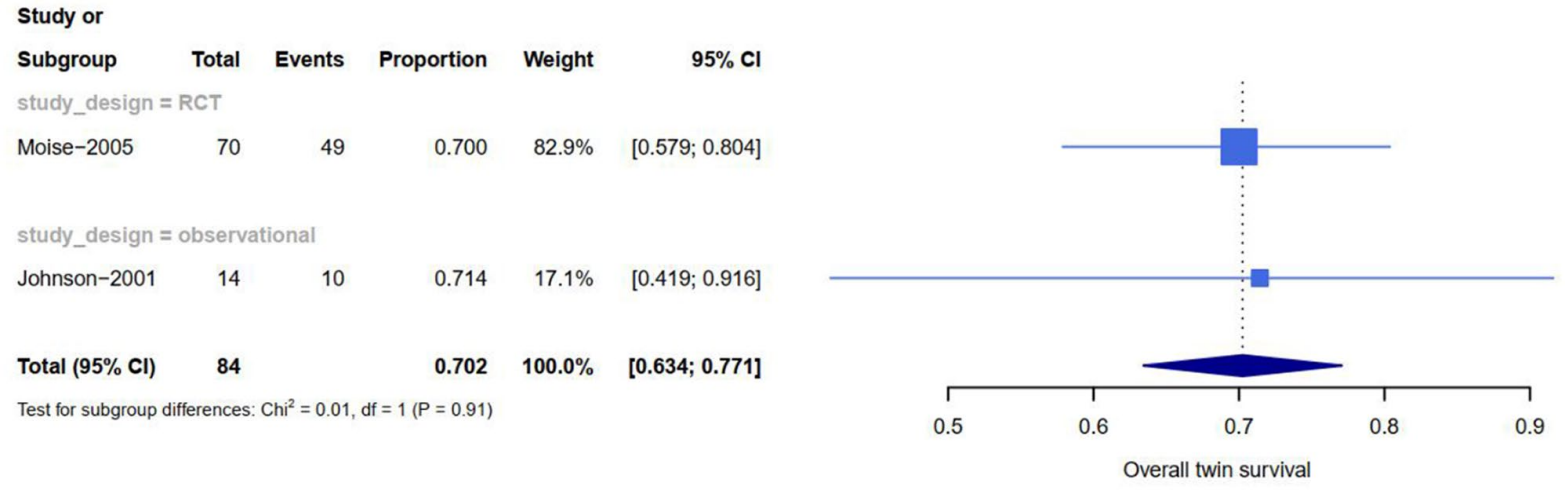
Forest plot showing at least on survival rates subdivided by type of studies of septostomy for TTTS

The rate of neonatal survival was 89% (95% CI 0.67-1) in twins managed expectantly, 70% (95% CI 0.63–0.77) in those who had septostomy, 69% (95% CI 0.65–0.73) in those who had laser, and 59% (95% CI 0.45–0.73) in those who had amnioreduction (see Table 1). Based on the meta-analysis, the highest rate of at least on survival were attributed to expected management (all cases in stage1). Additionally, 88 women (176 infant) were managed expectedly, the survival of both twins, one twin and at least one twin based on pregnancy (neonate) was 82% (81%), 15% (8%) and 97% (89%) respectively. The perinatal survival of both twins, one twin and at least one twin in patients treated with Fetoscopic laser based on pregnancy (neonate) was 56% (55%), 25 % (12%) and 81% (69%) respectively (see Figs. 3, 4, 5 and 6). Reports on complications after each modality were not readily available in all studies. All details about related complications and quality assessment are reported in the supplementary 3.  

**Table 1:** The rate of neonatal survival based on four modalities based on meta-analysis

Modality	One survival (%)	Double survival (%)	At least one survival (%)
**Fetoscopic laser**	12%	55%	69%
**Amnioreduction**	11%	42%	59%
**Septostomy**	10%	59%	70%
**Expected management**	8%	81%	89%

### Costs measurements

The mean cost for patients with TTTS under four treatment strategies is showed in Table 2. The highest cost rates were associated with the patients under treatment with amnioreduction (23 patients), septostomy (8 patients), Fetoscopic surgery (35 patients), and expected management (10 patients). Also, the cost of surgery and diagnostic medical services in all modalities was the highest. It is noticed to mention the diagnostic cost per individual in amnioreduction was the highest rate ($593.59).

**Table 2:** Direct medical costs of modalities to treat patients with TTTS ($)

Modality	Surgery	Laboratory	Medications	Physicians’ visits	Diagnostic medical services	Others	Total cost
Fetoscopic laser	444.19	31.42	50.25	72.62	223.27	49.74	871.51
Amnioreduction	659.47	80.22	362.37	59.65	593.59	265.50	2020.82
Septostomy	685.14	33.65	45.29	55.20	216.80	42.03	1178.14
Expected management	413.06	32.20	9.045	124.91	195.66	16.77	791.67

### Decision model analysis

**Fig. 7:**
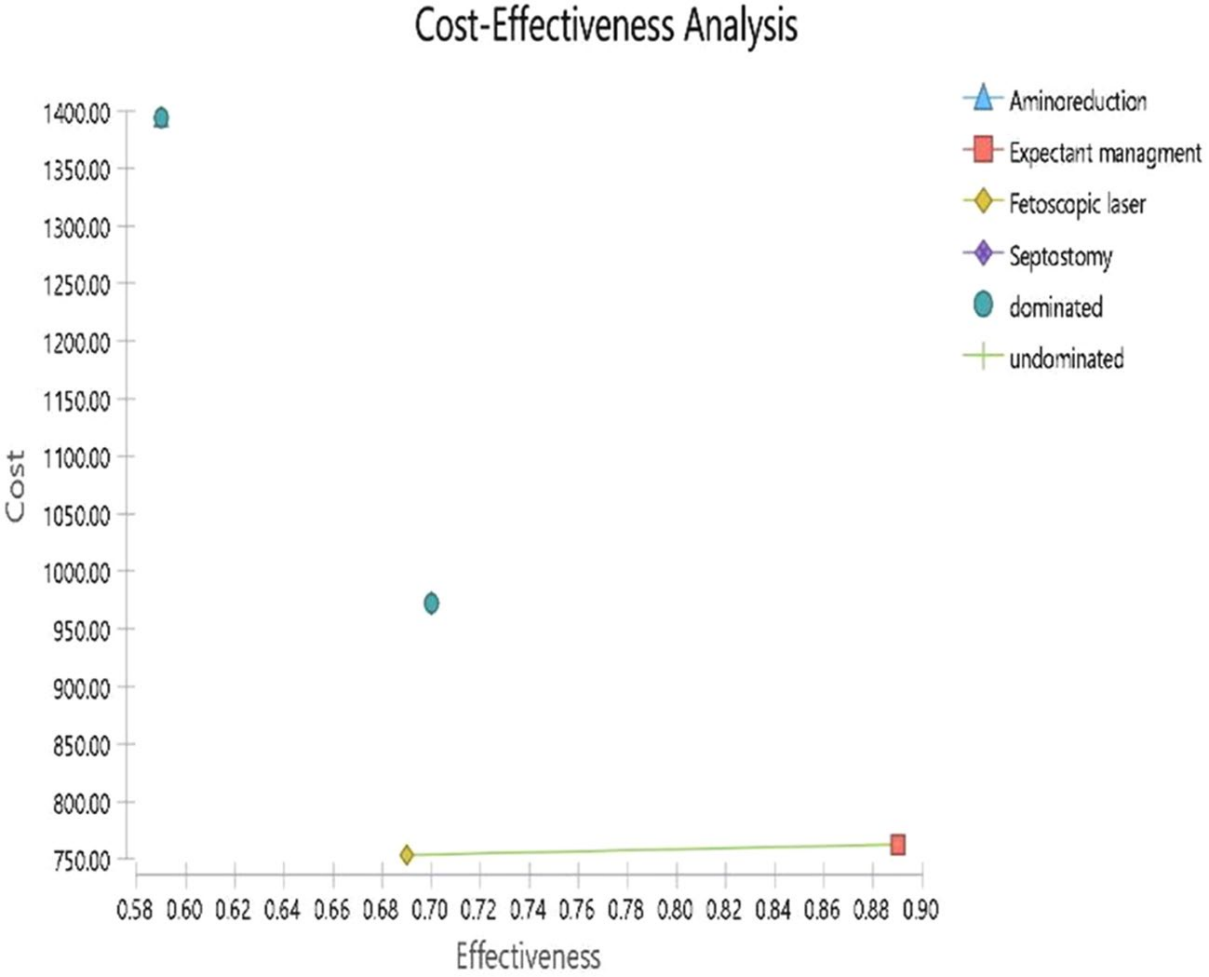
Cost effectiveness analysis for four treatment strategies

**Fig. 8:**
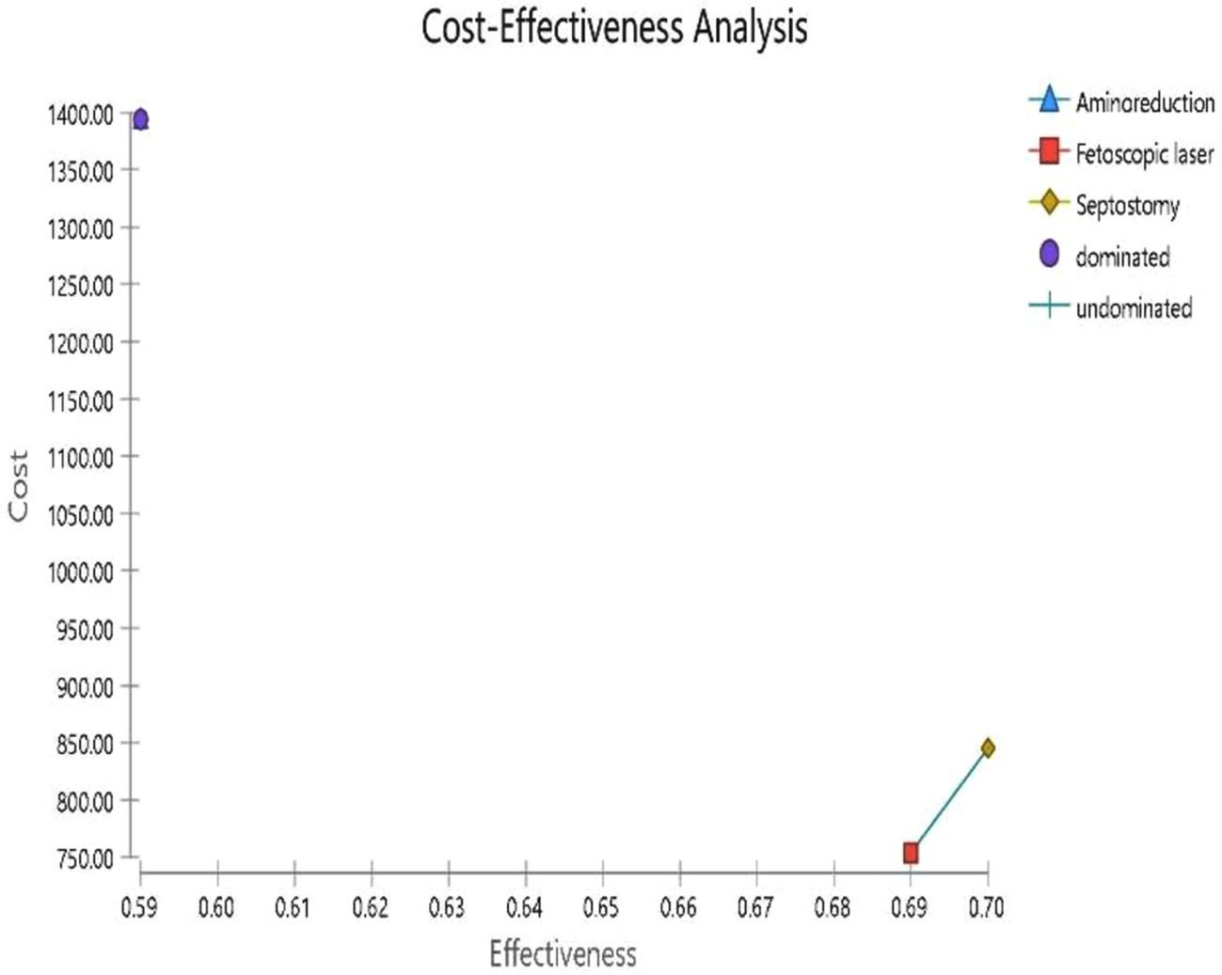
Cost effectiveness analysis after excluding expectant management strategy

As presented in Fig. 7, based on the cost-effectiveness analysis, expected management is the most cost-effective strategy for patients with TTTS. However, expected management is an appropriate strategy only in patients with TTTS in the early stages. Then, we conducted the analysis after excluding expected management strategy. Fetoscopic laser surgery, with the mean cost 871.46 $ and an overall survival rate of 0.69 considered the most cost-effectiveness strategy in other stages (see Fig. 8; Table 3).

**Table 3:** Cost-effectiveness analysis results

Strategy	Cost	Incr cost	Eff	Incr Eff	Incr C/E	NMB	C/E
Fetoscopic laser	753.4833	-	0.69	-	-	1148.157	1092.005
Expectant management	762.9185	9.4352	0.89	0.2	47.176	1689.922	857.2118
Septostomy	972.03	218.55	0.70	0.01	21875.18	957.16	1388.62
Amnioreduction	1393.472	639.9887	0.59	-0.1	-6399.89	232.568	2361.817

### One-way sensitivity analysis

**Fig. 9:**
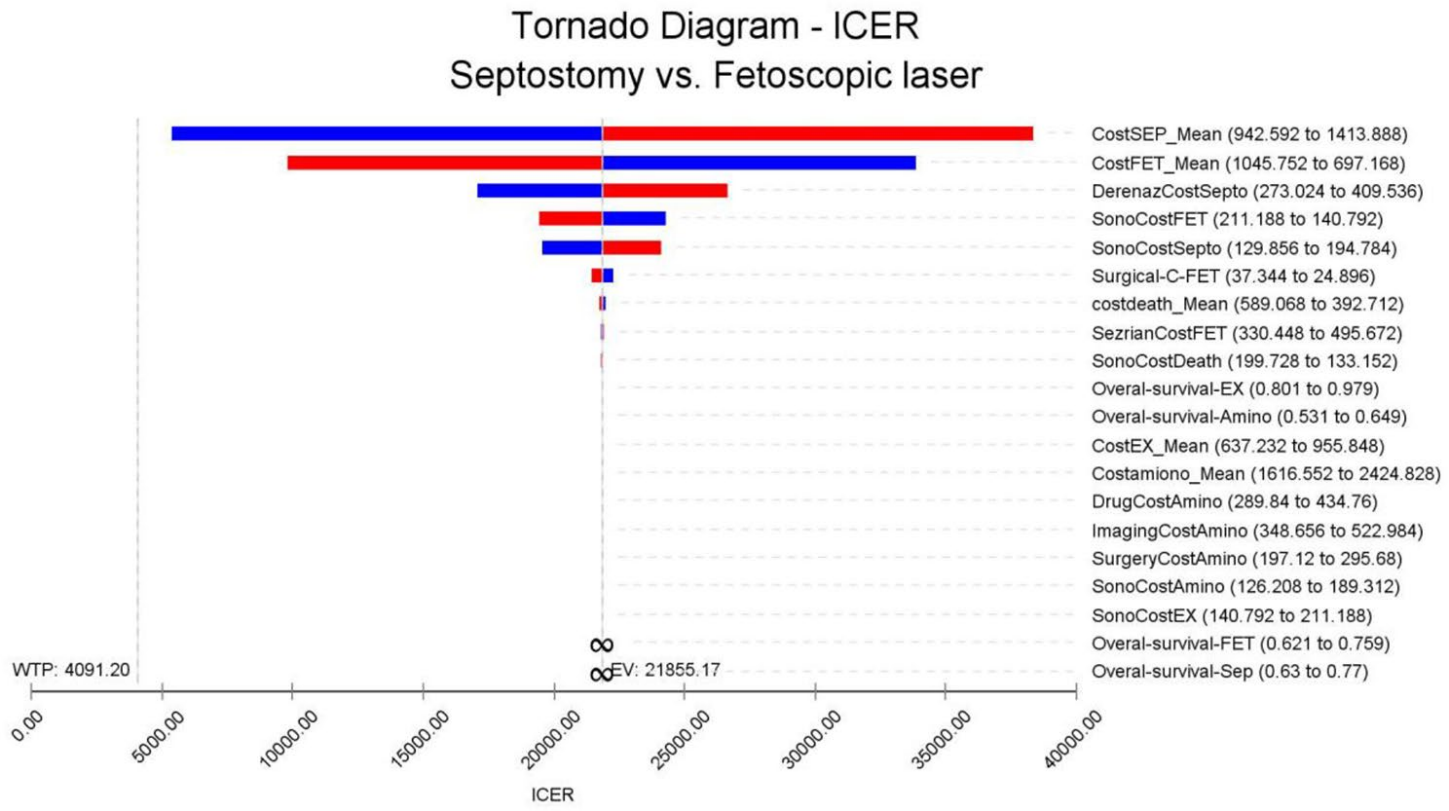
Tornado chart of the one-way deterministic analyses

According to the tornado diagram, ICER was most sensitive to the costs of septostomy and Fetoscopic laser. Therefore, a one-way sensitivity analysis was done on two variables. According to the analysis results, the result was not sensitive to changes in these variables (see Fig. 9).

### Probabilistic sensitivity analysis (PSA)

**Fig. 10:**
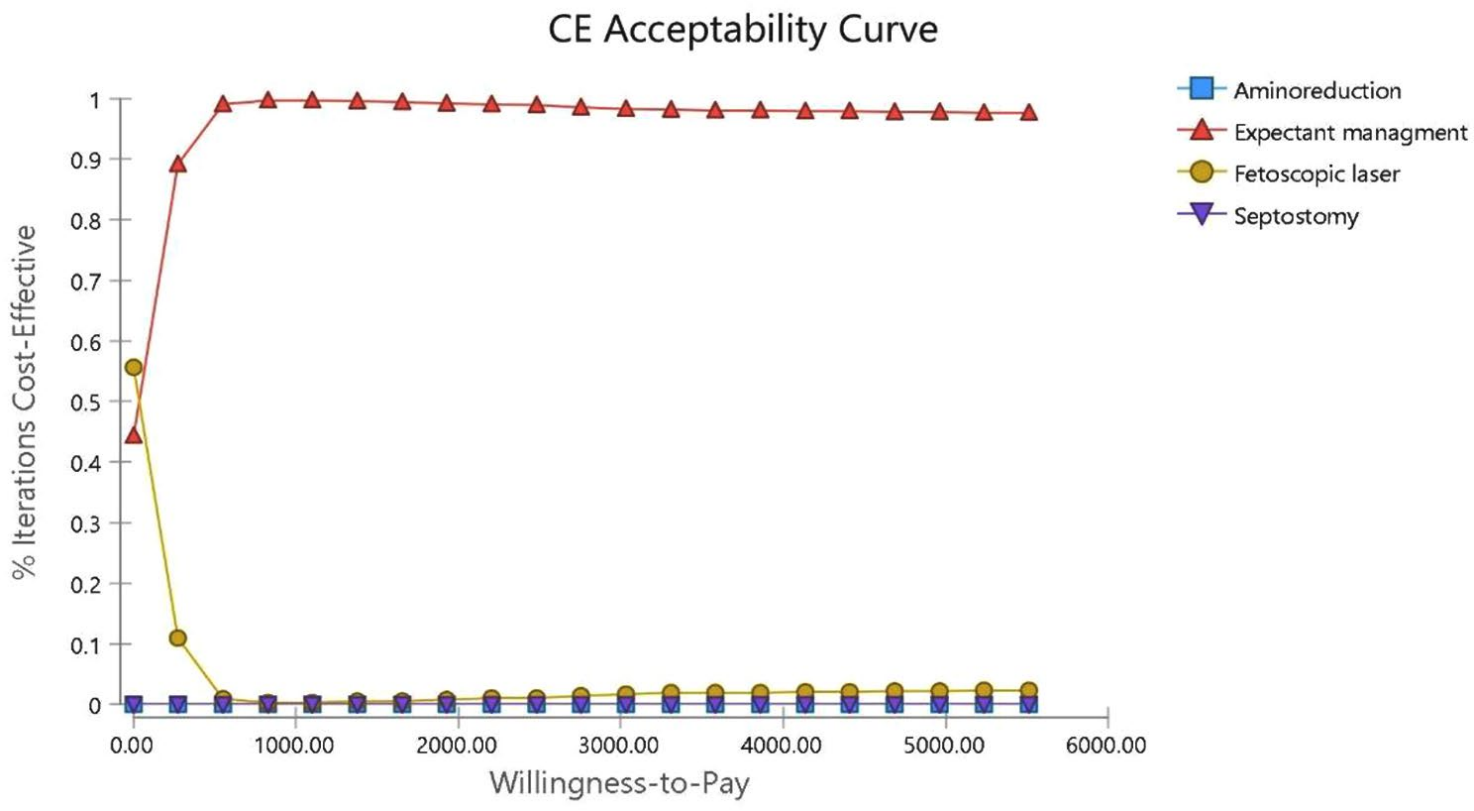
Monte Carlo Simulation cost effectiveness acceptability curve at WTP

**Fig. 11:**
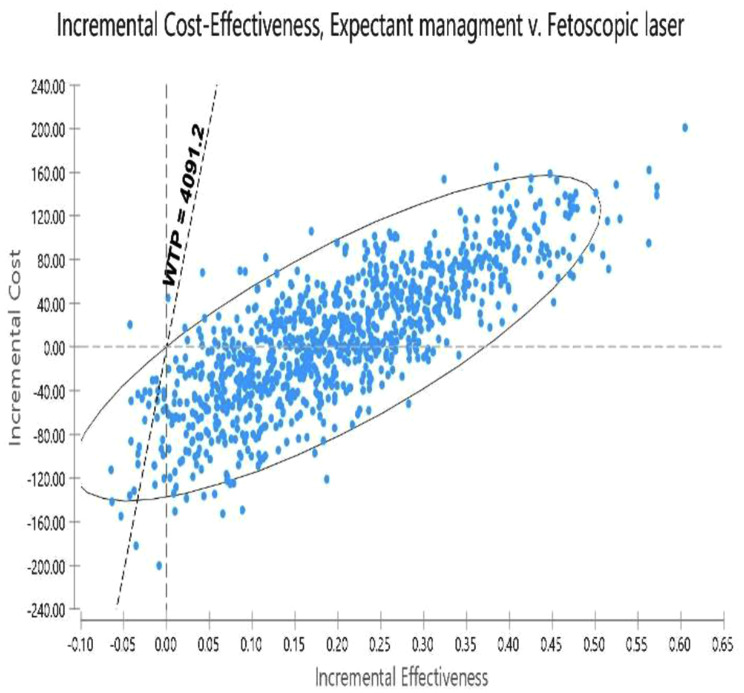
Scatter plot of PSA

**Fig. 12:**
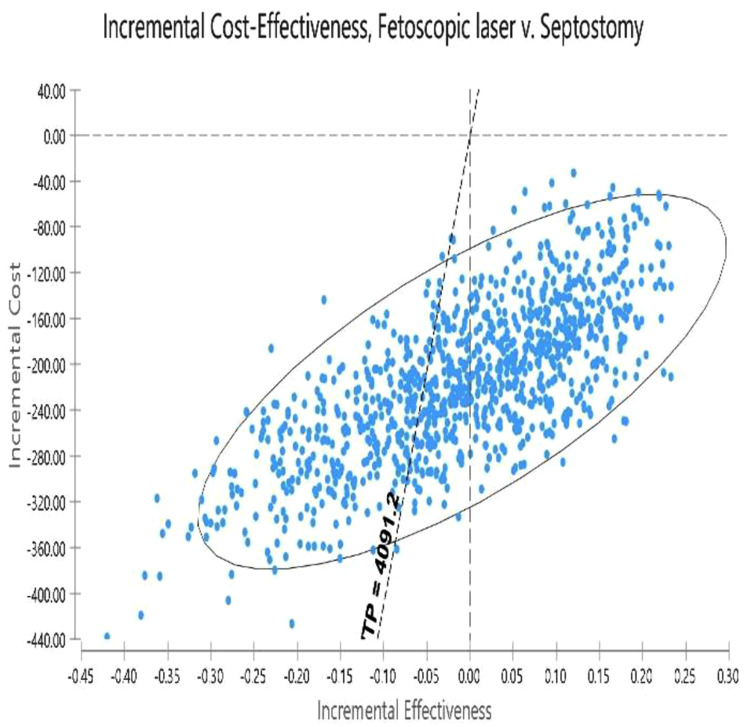
Scatter plot of PSA

The results of uncertainty measurement are presented using the cost-effectiveness acceptability curves and ICER scatter plot. The acceptability curves indicated that expectant management was the most cost-effective treatment in approximately 100% of simulations for the thresholds below $ 12273.6 (3 GDP per capita) (Fig. 10). The results on the scatter plot showed that expectant management was more effective and less costly in 55.8% of the cases. Although expectant management is highly costly in 40% of the cases, it is below the threshold of its comparator. When we compared Fetoscopic laser vs. septostomy, the results indicated lower cost in 42% of cases and higher effectiveness and lower cost below the threshold. Although Fetoscopic, in 45% of cases, was less effective and costly than septostomy, it is above the threshold. Then it is considered a non-cost-effective strategy (Figs. 11 and 12).

## Discussion

Based on our knowledge, this study is the first health-economic evaluation to compare Fetoscopic laser surgery with other modalities in treating patients with TTTS. Based on our results, Fetoscopic laser surgery is the most cost-effective therapy for patients with TTTS.

According to our meta-analysis results, the highest survival rate in TTTS belongs to the expected management strategy. Additionally, septostomy and Fetoscopic laser surgery had a similar survival rate (70%). Studies to manage TTTS pregnancies showed an overall survival rate of twins in the range of 86.4% after AR [15] and 70–88% after Fetoscopic surgery [7], and 77% with expectant managing patients [11].

Our analysis showed a significant difference in survival rate between Fetoscopic surgery and expectant management. Because of the low number of included studies (two studies) in the meta-analysis, this result can be a bit controversial. Also, Stirnemann et al., in a trial study, reported no difference in survival rate between them (80% in both groups) in patients with stage-1 TTTS. Then, they recommended that for women who have access to a surgical center within 48 h for immediate laser, expectant management is a reasonable option for stage-1 TTTS cases presenting before 26 weeks [11]. Based on our assessment, an immediate laser is still the best option. Based on the literature, more than half of patients with stage-1 TTTS will progress to higher stages throughout pregnancy and require rapid transfer to a surgical center [16]. For this reason, recent studies recommended that immediate laser should be still considered the best option.

In this respect, our results in the higher survival rate of at least one fetus in Fetoscopic laser are in line with the studies by Akkermans (2015) [7], Anh (2022) [17], Diehl (2017) [18]. Most studies on FLP for TTTS were limited to pregnancies treated before 26 weeks. Based on the results of a systematic review, expectant management, amnioreduction, laser surgery, and immediate delivery after diagnosis were used in twin pregnancies affected by late TTTS (after 26 weeks). They reported that in late TTTS, there is no consensus in managing patients. In other words, the optimal management for these pregnancies is yet to be ascertained. They concluded that less invasive palliative therapies such as amniodrainage or immediate delivery should be preferred as treatment options. In other words, recently, a few studies have reported the introduction of FLP for TTTS beyond 26 weeks of gestation. Nevertheless, there is still little evidence to perform FLP after 26 weeks of gestation [19]. A limitation of the evidence is that the number of cases is small. Then, the optimal treatment for TTTS with late presentation (after 27 weeks) is unknown.

In our analysis, amnioreduction had a lower survival rate (59%) than others. However, Gordon (2022) [15] reported that the controlled AR procedure resulted in a relatively high rate of twin survival (86%) with favorable long-term neurodevelopment outcomes. Therefore, the authors convinced that controlled AR procedures can be advised for managing TTTS pregnancies up to the 32nd week of gestation. At the same time, the study faces several significant limitations, like the absence of a control group and a small sample size.

We included two studies that had performed septostomy as a primary treatment option and reported a mean survival rate of 70%. While based on the recent evidence, septostomy has not been an advisable treatment for TTTS. Evidence of post-laser accidental septostomy shows an increased risk of adverse perinatal outcomes [20]. Therefore, they recommended effort should be made to prevent septostomy during laser therapy for TTTS.

Meanwhile, data on post-treatment complications such as TAPS, recurrent TTTS or PPROM, and miscarriage were often unavailable in the reported studies. However, based on the reported data, PROM, repeated TTTS, and TAPS are generally assumed to be the most common complication after laser therapy [21]. Furthermore, technological advancements and innovations in laser procedures have led to increased perinatal survival and a decreased incidence of neonatal complications, including brain injury and neurodevelopmental impairment.

Based on our results, costs of expected management and Fetoscopic surgery were less than amnioreduction and septostomy. The reasons for such a difference could be related to the higher need for follow-up in patients under amnioreduction and septostomy compared to Fetoscopic laser and their need to use more imaging services and physician’s visits. However, the cost of the four modalities was lower than the threshold. Most of the costs used for the analysis are based on government tariffs. Because of the high inflation rate in Iran, when we exchange the prices from Iranian Rials (IRR) to US Dollars (USD), its value would be lower than the results of other countries.

Based on the results of the decision model analysis, expected management had the lowest cost ($ 791.67) and the highest rate in at least one survival (89%) than others (Fetoscopic surgery, amnioreduction, and septostomy) in the early stages. Therefore, because of lower cost and higher survival rate, expected management in patients with TTTS in stage 1 was considered the cost-effective option. Although it is noteworthy, the outcome is extracted based on two studies. The evidence for treating early stages of TTTS without invasive treatment strategies due to the percentage of progressing to higher stages remains controversial. For this reason, we again conducted the decision model analysis without the expected management strategy. Our result showed that Fetoscopic surgery had a lower cost and higher survival rate in TTTS than others (amnioreduction and septostomy). The evidence for Fetoscopic surgery is robust compared to others. Of the lack of data, we could not calculate the cost of post-complications after the modalities for treating TTTS. To compare with the threshold of effectiveness in economic analyses, all modalities would be highly cost-effective.

Despite the effectiveness of Fetoscopic surgery in a wide range of studies, in the country, there is only one center to perform this surgery. The results of this analysis support effort to increase the number of centers that started to perform this procedure by opening regional centers for Fetoscopic surgery. Increased awareness of clinicians may have resulted in improved timely referral and a decreasing number of cases with advanced disease and poor outcomes.

The results of the one-way sensitivity analysis showed that the model was sensible to the cost of septostomy and the Fetoscopic surgery. With variations to the parameters, the ICER of Fetoscopic surgery compared with septostomy remained cost-effective.

This study has some limitations. First, we have not considered different techniques in the treatment modalities, especially for the Fetoscopic surgery. We only focused on the direct medical cost during the pregnancy period. Also, we did not consider the cost of post-complications after modalities. Consequently, they could not be captured by the model. Meanwhile, our analysis did not consider long-term outcomes such as severe mental impairment and delayed psychomotor development as complications. We did not consider different stages in our model, because of non-reporting related data in most of the studies. Then we could not compare the results between expected management modality to other modalities.

In conclusion, our model found Fetoscopic laser surgery in all stages is the most cost-effective therapy for patients with TTTS. Fetoscopic laser surgery thus should be considered a cost-effective choice to treating TTTS. The results of our findings could be helpful in decisions regarding third-party coverage and establishing more regional referral centers for TTTS treatment.

### Electronic supplementary material

Below is the link to the electronic supplementary material.

Supplementary Material 1

## Data Availability

All data analyzed during this study are included in the article and its supplementary information files. Any further information is available from the corresponding author on reasonable request.

## Abbreviations

TTTS: Twin-twin transfusion syndrome
ICER: Incremental cost-effectiveness ratio
PSA: A probabilistic sensitivity analysis
IUFD: Intrauterine fetal demise
DMC: Direct medical costs
PPROM: Preterm premature rupture of membranes
TAPS: Twin anemia-polycythemia sequence
WTP: Willingness to pay
IRR: Iranian Rial
