# Supplementary material for: Fetoscopic laser versus amnioreduction, septostomy, and expected management for the treatment of twin-twin transfusion syndrome (TTTS): an economic evaluation analysis in Iran
Source: Cost Eff Resour Alloc. 2024 May 9;22:39. doi: 10.1186/s12962-024-00551-2 (PMC11080211; doi:10.1186/s12962-024-00551-2)
Supplement: Supplementary file 1 — Supplementary Material 1 [file 12962_2024_551_MOESM1_ESM.docx]

**Supplementary1: search strategies**

**Keywords**

- Survival OR perinatal survival
- twin-twin
- (twin near transfusion)
- twin-twin transfusion syndrome
- TTTS
- Pregnancy Reduction
- monochorionic pregnancy*
- [monochorionic twin](https://www.tandfonline.com/keyword/Monochorionic+Twins)*
- Fetofetal Transfusion
- [Laser OR Laser surgery](https://www.tandfonline.com/keyword/Laser+Surgery) OR laser therapy OR sequential laser OR selective laser
- ((laser next ablation) and twin-twin)
- ((laser next ablation) and TTTS)
- laser coagulation
- endoscopic laser coagulation
- Fetoscopic laser surgery
- (Fetoscopic near TTTS)
- (Fetoscopic near twin-twin)
- [septostomy](https://pubmed.ncbi.nlm.nih.gov/32959414/)
- selective feticide
- ((laser next coagulation) and TTTS)
- Amnioreduction OR amniodrainage OR serial amniocentesis
- (Amnioreduction near [septostomy](https://pubmed.ncbi.nlm.nih.gov/32959414/))

| PubMed search (2022/06/12) | |
| --- | --- |
| "twin-twin"[Title/Abstract] | 1325 |
| "twin-twin transfusion syndrome"[Title/Abstract] | 1169 |
| "Fetofetal transfusion"[MeSH Terms] | 2270 |
| "TTTS"[Title/Abstract] | 1036 |
| "Pregnancy Reduction"[Title/Abstract] | 341 |
| "monochorionic pregnancy*"[Title/Abstract] | 83 |
| "monochorionic twin*"[Title/Abstract] | 1411 |
| "twin-twin"[Title/Abstract] OR "twin-twin transfusion syndrome"[Title/Abstract] OR "Fetofetal transfusion"[MeSH Terms] OR "TTTS"[Title/Abstract] OR "Pregnancy Reduction"[Title/Abstract] OR "monochorionic pregnancy*"[Title/Abstract] OR "monochorionic twin*"[Title/Abstract] | 3820 |
| ("Laser"[Title/Abstract] OR "Laser surgery"[Title/Abstract] OR "laser therapy"[Title/Abstract] OR "sequential laser"[Title/Abstract] OR "selective laser"[Title/Abstract]) AND "selective laser"[All Fields]) | 2326 |
| ((laser AND ablation[Title/Abstract]) AND twin-twin[Title/Abstract]) | 97 |
| ((laser[Title/Abstract] AND ablation[Title/Abstract]) AND "TTTS"[Title/Abstract]) | 118 |
| "laser coagulation"[Title/Abstract] | 1351 |
| "endoscopic laser coagulation"[Title/Abstract] | 56 |
| "Fetoscopic laser"[Text Word] | 465 |
| (Fetoscopic[Title/Abstract] AND TTTS[Title/Abstract]) | 329 |
| ((Fetoscopic[Title/Abstract] AND twin-twin[Title/Abstract])) AND ((Fetoscopic AND "twin-twin"[Title/Abstract])) | 282 |
| "septostomy"[Text Word] | 1017 |
| "selective feticide"[Text Word] | 142 |
| ((laser[Title/Abstract] AND coagulation[Title/Abstract]) AND TTTS[Title/Abstract]) | 145 |
| "Amnioreduction"[Text Word] OR "amniodrainage"[Text Word] OR "serial amniocentesis"[Text Word] | 302 |
| "amniocentesis"[MeSH Terms] | 8096 |
| (Amnioreduction[Title/Abstract] AND septostomy[Title/Abstract]) | 30 |
| "Laser"[Title/Abstract] OR "Laser surgery"[Title/Abstract] OR "laser therapy"[Title/Abstract] OR "sequential laser"[Title/Abstract] OR ("selective laser"[Title/Abstract] AND "selective laser"[All Fields]) OR "laser coagulation"[Title/Abstract] OR "endoscopic laser coagulation"[Title/Abstract] OR "Fetoscopic laser"[Text Word] OR "septostomy"[Text Word] OR ("selective feticide"[Title/Abstract] AND "selective feticide"[Text Word]) OR ("Amnioreduction"[Text Word] OR "amniodrainage"[Text Word] OR "serial amniocentesis"[Text Word]) OR "amniocentesis"[MeSH Terms] | 303441 |
| ("Laser"[Title/Abstract] OR "Laser surgery"[Title/Abstract] OR "laser therapy"[Title/Abstract] OR "sequential laser"[Title/Abstract] OR ("selective laser"[Title/Abstract] AND "selective laser"[All Fields]) OR "laser coagulation"[Title/Abstract] OR "endoscopic laser coagulation"[Title/Abstract] OR "Fetoscopic laser"[Text Word] OR "septostomy"[Text Word] OR ("selective feticide"[Title/Abstract] AND "selective feticide"[Text Word]) OR ("Amnioreduction"[Text Word] OR "amniodrainage"[Text Word] OR "serial amniocentesis"[Text Word]) OR "amniocentesis"[MeSH Terms]) AND ("twin-twin"[Title/Abstract] OR "twin-twin transfusion syndrome"[Title/Abstract] OR "Fetofetal transfusion"[MeSH Terms] OR "TTTS"[Title/Abstract] OR "Pregnancy Reduction"[Title/Abstract] OR "monochorionic pregnancy*"[Title/Abstract] OR "monochorionic twin*"[Title/Abstract]) | 1158 |

| Cochrane search (2022/06/12) | |
| --- | --- |
| 1. ("twin-twin"):ti,ab,kw | 74 |
| 2.("twin-twin transfusion syndrome"):ti,ab,kw | 65 |
| 3.MeSH descriptor: [Fetofetal Transfusion] explode all trees | 25 |
| 4."TTTS"[Title/Abstract] | 35 |
| 5.("Pregnancy Reduction"):ti,ab,kw | 21 |
| 6.("monochorionic pregnancy"):ti,ab,kw | 5 |
| 7.("monochorionic twin*"):ti,ab,kw | 44 |
| #1 OR #2 OR #3 OR #4 OR #5 OR #6 OR #7 | 137 |
| 9.((Laser OR "laser surgery" OR "laser therapy" OR "sequential laser" OR "selective laser")):ti,ab,kw | 21339 |
| 10.(((laser next ablation) and twin-twin)):ti,ab,kw | 2 |
| 11.((laser next ablation) and TTTS) | 1 |
| 12.("laser coagulation"):ti,ab,kw | 1205 |
| 13.("endoscopic laser coagulation"):ti,ab,kw | 3 |
| 14.(Fetoscopic laser):ti,ab,kw | 46 |
| 15.((Fetoscopic near TTTS)):ti,ab,kw | 7 |
| 16.((Fetoscopic near twin-twin)):ti,ab,kw | 13 |
| 17.MeSH descriptor: [Fetoscopy] explode all trees | 24 |
| 18.(septostomy):ti,ab,kw | 49 |
| 19.(selective feticide):ti,ab,kw | 10 |
| 20.(((laser next coagulation) and TTTS)):ti,ab,kw | 16 |
| 21.(Amnioreduction OR amniodrainage OR serial amniocentesis):ti,ab,kw | 21 |
| 22.MeSH descriptor: [Amniocentesis] explode all trees | 129 |
| 23.(Amnioreduction near [septostomy](https://pubmed.ncbi.nlm.nih.gov/32959414/)) |  |
| #9 OR #10 OR #11 OR #12 OR #13 OR #14 OR #15 OR #16 OR #17 OR #18 OR #19 OR #20 OR #21 OR #22 OR #23 | 21534 |
| #8 AND #24 | 61 |

| Scopus (2022/06/12) | |
| --- | --- |
| TITLE-ABS-KEY (twin-twin) | 3035 |
| TITLE-ABS-KEY ( "twin-twin transfusion syndrome" ) | 2054 |
| TITLE-ABS-KEY ( "Fetofetal Transfusion" ) | 1946 |
| TITLE-ABS-KEY ( " TTTS" ) | 1240 |
| TITLE-ABS-KEY (" Pregnancy Reduction") | 941 |
| TITLE-ABS-KEY ("monochorionic pregnancy") | 346 |
| TITLE-ABS-KEY ("monochorionic twin") | 1816 |
| TITLE-ABS-KEY ( ( "twin-twin" )  OR  ( "twin PRE/2 transfusion" )  OR  ( "twin-twin transfusion syndrome" )  OR  ( "TTTS" )  OR  ( "Pregnancy Reduction" )  OR  ( "monochorionic pregnancy*" )  OR  ( "monochorionic twin*" )  OR  ( "Fetofetal Transfusion" ) ) | 5668 |
| TITLE (("laser surgery" OR "laser therapy" OR "sequential laser" OR "selective laser")) | 16078 |
| TITLE-ABS-KEY ( ( ( laser  AND  ablation )  AND  twin-twin ) ) | 119 |
| TITLE-ABS-KEY ( ( ( laser AND ablation ) AND "TTTS" ) ) | 149 |
| TITLE-ABS-KEY  ("laser coagulation") | 24980 |
| TITLE-ABS-KEY  ("endoscopic laser coagulation") | 69 |
| TITLE-ABS-KEY  (Fetoscopic laser) | 647 |
| TITLE-ABS-KEY  ((Fetoscopic near/2 TTTS)) | 1 |
| TITLE-ABS-KEY  ((Fetoscopic near/2 twin-twin)) | 3 |
| TITLE-ABS-KEY (Fetoscopy) | 2734 |
| TITLE-ABS-KEY  (septostomy) | 1,396 |
| TITLE-ABS-KEY  (selective feticide) | 230 |
| TITLE-ABS-KEY  (((laser and coagulation) and TTTS)) | 408 |
| TITLE-ABS-KEY  (Amnioreduction OR amniodrainage OR serial amniocentesis) | 380 |
| TITLE-ABS-KEY  (Amnioreduction near [septostomy](https://pubmed.ncbi.nlm.nih.gov/32959414/)) | 45 |
| TITLE-ABS-KEY  ((("laser surgery" OR "laser therapy" OR "sequential laser" OR "selective laser")) OR ( ( ( laser  AND  ablation )  AND  "twin-twin" ) ) OR ( ( ( laser AND ablation ) AND "TTTS" ) ) OR ("laser coagulation") OR ("endoscopic laser coagulation") OR (Fetoscopic laser) OR ((Fetoscopic near/2 TTTS)) OR ((Fetoscopic near/2 "twin-twin")) OR (Fetoscopy)  OR (septostomy) OR (selective feticide) OR (((laser AND coagulation) and TTTS)) OR (Amnioreduction OR amniodrainage OR serial amniocentesis) OR (Amnioreduction AND [septostomy](https://pubmed.ncbi.nlm.nih.gov/32959414/))) AND (( ( "twin-twin" )  OR  ( "twin AND transfusion" )  OR  ( "twin-twin transfusion syndrome" )  OR  ( "TTTS" )  OR  ( "Pregnancy Reduction" )  OR  ( "monochorionic pregnancy*" )  OR  ( "monochorionic twin*" )  OR  ( "Fetofetal Transfusion" ))) | 1584 |

| Google Scholar |  |
| --- | --- |
| (("twin-twin") OR ("twin-twin transfusion syndrome") OR ("Fetofetal transfusion") OR ("TTTS") OR ("Pregnancy Reduction") OR ("monochorionic pregnancy*") OR ("monochorionic twin*")) AND (([Laser OR “Laser surgery](https://www.tandfonline.com/keyword/Laser+Surgery)” OR “laser therapy” OR “sequential laser” OR “selective laser”) OR ((“laser next ablation”) and “twin-twin”) OR ((“laser next ablation”) and “TTTS”) OR (“laser coagulation”) OR (“endoscopic laser coagulation”) OR (“Fetoscopic laser surgery”) OR (Fetoscopic near TTTS) OR (Fetoscopic near twin-twin) OR (“[septostomy](https://pubmed.ncbi.nlm.nih.gov/32959414/)”) OR (“selective feticide”) OR ((laser next coagulation) and TTTS) OR (Amnioreduction OR amniodrainage OR serial amniocentesis) OR (Amnioreduction near [septostomy](https://pubmed.ncbi.nlm.nih.gov/32959414/))):ti, ab, kw | 149 |

| WEB of science (2022/06/12) | |
| --- | --- |
| TS= ( ( "twin-twin" )  OR  ( "twin PRE/2 transfusion" )  OR  ( "twin-twin transfusion syndrome" )  OR  ( "TTTS" )  OR  ( "Pregnancy Reduction" )  OR  ( "monochorionic pregnancy*" )  OR  ( "monochorionic twin*" )  OR  ( "Fetofetal Transfusion" ) ) | 4688 |
| TS= ((("laser surgery" OR "laser therapy" OR "sequential laser" OR "selective laser")) OR ( ( ( laser  AND  ablation )  AND  "twin-twin" ) ) OR ( ( ( laser AND ablation ) AND "TTTS" ) ) OR ("laser coagulation") OR ("endoscopic laser coagulation") OR (Fetoscopic laser) OR ((Fetoscopic near/2 TTTS)) OR ((Fetoscopic near/2 "twin-twin")) OR (Fetoscopy)  OR (septostomy) OR (selective feticide) OR (((laser AND coagulation) and TTTS)) OR (Amnioreduction OR amniodrainage OR serial amniocentesis) OR (Amnioreduction AND [septostomy](https://pubmed.ncbi.nlm.nih.gov/32959414/))) AND (( ( "twin-twin" )  OR  ( "twin AND transfusion" )  OR  ( "twin-twin transfusion syndrome" )  OR  ( "TTTS" )  OR  ( "Pregnancy Reduction" )  OR  ( "monochorionic pregnancy*" )  OR  ( "monochorionic twin*" )  OR  ( "Fetofetal Transfusion" ))) | 1276 |
| ( ((("laser surgery" OR "laser therapy" OR "sequential laser" OR "selective laser")) OR ( ( ( laser AND ablation ) AND "twin-twin" ) ) OR ( ( ( laser AND ablation ) AND "TTTS" ) ) OR ("laser coagulation") OR ("endoscopic laser coagulation") OR ("Fetoscopic laser") OR (("Fetoscopic" AND "TTTS")) OR (("Fetoscopic" AND "twin-twin")) OR (Fetoscopy) OR (septostomy) OR (selective feticide) OR ((("laser" AND "coagulation") and "TTTS")) OR (Amnioreduction OR amniodrainage OR serial amniocentesis) OR (Amnioreduction AND septostomy)) )) AND TS=(( ( "twin-twin" ) OR ( ("twin") AND ("transfusion") ) OR ( "twin-twin transfusion syndrome" ) OR ( "TTTS" ) OR ( "Pregnancy Reduction" ) OR ( "monochorionic pregnancy*" ) OR ( "monochorionic twin*" ) OR ( "Fetofetal Transfusion" ) ) | 1483 |

**Supplementary 2: included studies characteristics**

| **Table 1: included studies** | | | | | | | | |
| --- | --- | --- | --- | --- | --- | --- | --- | --- |
| **row** | **Study (Author-year)** | **title** | **start (year)** | **end (year)** | **type of methods** | **country** | **patients** | **modality** |
| 1 | Huber-2006 | Stage-Related Outcome in Twin–Twin Transfusion Syndrome Treated by Fetoscopic Laser Coagulation | 1999 | 2003 | Observational |  | TTTS | Fetoscopic-laser |
| 2 | Kowitt-2012 | Long-term morbidity after fetal endoscopic surgery for severe twin-to-twin transfusion syndrome | 2000 | 2009 | Observational | USA | TTTS | Fetoscopic-laser |
| 3 | Anh-2021 | Fetoscopic Laser Ablation for the Selective Fetal Reduction in Twin-Twin Transfusion Syndrome Stage II–IV: The Experience of a New Fetal Medicine Center | 2019 | 2021 | observational |  | TTTS | Fetoscopic-laser |
| 4 | Anh-2022 | The Efficacy of Fetoscopic Laser Surgery in Twin-Twin Transfusion Syndrome: A Preliminary Vietnamese Study | 2019 | 2021 | Observational | Vietnam | TTTS | Fetoscopic-laser |
| 5 | Bamberg-2021 | Neither the differentiation between twin–twin transfusion syndrome Stages I and II nor III and IV makes a difference regarding the probability of double survival after laser therapy | 1995 | 2013 | Observational |  | TTTS | Fetoscopic-laser |
| 6 | Bartin-2022 | Selective vs complete fetoscopic coagulation of vascular equator: a matched comparative study | 2006 | 2020 | observational |  | TTTS | Fetoscopic-laser |
| 7 | Bergh-2020 | Pregnancy outcomes associated with chorioamnion membrane separation severity following fetoscopic laser surgery for twin-twin transfusion syndrome | 2011 | 2018 | Observational | USA | TTTS | Fetoscopic-laser |
| 8 | Chang-2012(1) | Short-term outcomes of fetoscopic laser surgery for severe twinetwin transfusion syndrome from Taiwan single center experience: Demonstration of learning curve effect on the fetal outcomes | 2005 | 2010 | Observational | Taiwan | TTTS | Fetoscopic-laser |
| 9 | Chang-2012(2) | The neurological outcomes of surviving twins in severe twin–twin transfusion syndrome treated by fetoscopic laser photocoagulation at a newly established center | 2005 | 2010 | Observational | Taiwan | TTTS | Fetoscopic-laser |
| 10 | Chmait-2010 | Perinatal survival following preferential sequential selective laser surgery for twin-twin transfusion syndrome | 2006 | 2008 | observational | usa | TTTS | Fetoscopic-laser |
| 11 | Chmait-2011 | Stage-based outcomes of 682 consecutive cases of twin–twin transfusion syndrome treated with laser surgery: the USFetus experience | 2002 | 2010 | Observational | USA | TTTS | Fetoscopic-laser |
| 12 | Chmait-2015(2) | Fetal brain-sparing after laser surgery for twin-twin transfusion syndrome appears associated with two-year neurodevelopmental outcomes† | 2008 | 2010 | Observational | USA | TTTS | Fetoscopic-laser |
| 13 | Chmait-2017 | Neonatal cerebral lesions predict 2-year neurodevelopmental impairment in children treated with laser surgery for twin–twin transfusion syndrome | 2007 | 2010 | Observational | USA | TTTS | Fetoscopic-laser |
| 14 | Crombleholme-2007 | A prospective, randomized, multicenter trial of amnioreduction vs selective fetoscopic laser photocoagulation for the treatment of severe twin-twin transfusion syndrome |  |  | RCT |  | TTTS | Fetoscopic-laser&Amnioreduction |
| 15 | Diehl-2017 | Fetoscopic laser coagulation in 1020 pregnancies with twin-to-twin transfusion syndrome demonstrates improvement of double survival rates | 1995 | 2013 | Observational | Germany | TTTS | Fetoscopic-laser |
| 16 | Duy-Anh-2022 | The Efficacy of Fetoscopic Laser Surgery in Twin-Twin Transfusion Syndrome: A Preliminary Vietnamese Study | 2019 | 2021 | Observational | Vietnam | TTTS | Fetoscopic-laser |
| 17 | Fang-2019 | Effects and outcomes of septostomy in twin-to-twin transfusion syndrome after fetoscopic laser therapy | 2005 | 2018 | Observational | Taiwan | TTTS | Septostomy&fetoscpic laser |
| 18 | Gordon-2017 | Incidental Septostomy after Laser Surgery for Twin-Twin Transfusion Syndrome: Perinatal Outcomes and Antenatal Management | 2006 | 2015 | Observational | USA | TTTS | Fetoscopic-laser |
| 19 | Gordon-2022 | Controlled amnioreduction for twin-to-twin transfusion syndrome | 2004 | 2015 | RCT |  | TTTS | Amnioreduction |
| 20 | Gray-2006 | Perinatal Outcomes With Laser Surgery for Twin–Twin Transfusion Syndrome |  |  | observational |  | TTTS | Fetoscopic-laser&Amnioreduction |
| 21 | Gray-2011 | Neurodevelopmental outcome and risk factors for disability for twin-twin transfusion syndrome treated with laser surgery | 2002 | 2006 | Observational | Australia | TTTS | Fetoscopic-laser |
| 22 | Habli-2009 | Incidence of complications in twin-twin transfusion syndrome after selective fetoscopic laser photocoagulation: a single-center experience | 2005 | 2008 | Observational | USA | TTTS | Fetoscopic-laser |
| 23 | Has-2014 | Stage-Related Outcome after Fetoscopic Laser Ablation in Twin-to-Twin Transfusion Syndrome | 2006 | 2013 | Observational | Turkey | TTTS | Fetoscopic-laser |
| 24 | Hecher-2000 | Endoscopic laser coagulation of placental anastomoses in 200 pregnancies with severe mid-trimester twin-to-twin transfusion syndrome | 1995 | 1999 | observational |  | TTTS | Fetoscopic-laser |
| 25 | Herberg-2005 | Long term cardiac follow up of severe twin to twin transfusion syndrome after intrauterine laser coagulation |  |  | Observational | Germany | TTTS | Fetoscopic-laser |
| 26 | Ierullo-2007 | Severe twin–twin transfusion syndrome: outcome after fetoscopic laser ablation of the placental vascular equator | 2002 | 2006 | Observational | UK | TTTS | Fetoscopic-laser |
| 27 | Johnson-2001 | Amnioreduction versus septostomy in twin-twin transfusion syndrome | 1998 | 2000 | Observational | USA | TTTS | Amnioreduction&septostomy |
| 28 | Kempen-2016 | Increased Risk Of Early-Onset Neonatal Sepsis After Laser Surgery For Twin-to-Twin Transfusion Syndrome | 2002 | 2015 | observational |  | TTTS | Fetoscopic-laser |
| 29 | Klink-2014 | Improvement in neurodevelopmental outcome in survivors of twin-twin transfusion syndrome treated with laser surgery | 2000 | 2010 | Observational | Netherlands | TTTS | Fetoscopic-laser |
| 30 | Klink-2015 | Neurodevelopmental outcome at 2 years in twin-twin transfusion syndrome survivors randomized for the Solomon trial. |  |  | RCT | (Italy AND Netherlands | TTTS | Fetoscopic-laser |
| 31 | Knijnenburg-2021 | Placental Abruption after Fetoscopic Laser Surgery in Twin-Twin Transfusion Syndrome: The Role of the Solomon Technique | 2002 | 2020 | observational |  | TTTS | Fetoscopic-laser |
| 32 | Korsakissok-2018 | Mortality, morbidity and 2-years neurodevelopmental prognosis of twin to twin transfusion syndrome after fetoscopic laser therapy: a prospective, 58 patients cohort study | 2008 | 2014 | Observational |  | TTTS | Fetoscopic-laser |
| 33 | Loh-2020 | Outcomes following selective fetoscopic laser ablation for twin-to-twin transfusion syndrome: a single-centre experience | 2011 | 2014 | Observational |  | TTTS | Fetoscopic-laser |
| 34 | Lombardo-2011 | Laser Ablation of Placental Vessels in Twin-to-Twin Transfusion Syndrome: A Paradigm for Endoscopic Fetal Surgery | 2000 | 2010 | Observational |  | TTTS | Fetoscopic-laser |
| 35 | Lopriore-2005 | Incidence, origin, and character of cerebral injury in twin-to-twin transfusion syndrome treated with fetoscopic laser surgery | 2002 | 2005 | Observational | Netherlands | TTTS | Fetoscopic-laser |
| 36 | Lopriore-2006 | Congenital Heart Disease in Twin-to-twin Transfusion Syndrome Treated with Fetoscopic Laser Surgery | 2002 | 2005 | Observational |  | TTTS | Fetoscopic-laser |
| 37 | Lopriore-2007(1) | Long-term neurodevelopmental outcome in twin-to-twin transfusion syndrome treated with fetoscopic laser surgery | 2000 | 2003 | Observational | Netherlands | TTTS | Fetoscopic-laser |
| 38 | Lopriore-2007(2) | Preterm premature rupture of membranes after fetoscopic laser surgery for twin-twin transfusion syndrome | 2011 | 2014 | Observational | USA | TTTS | Fetoscopic-laser |
| 39 | Mari-2000 | Long-term outcome in twin-twin transfusion syndrome treated with serial aggressive amnioreduction |  |  | Observational |  | TTTS | Amnioreduction |
| 40 | Martinez-2011 | Incidence and clinical implications of early inadvertent septostomy after laser therapy for twin–twin transfusion syndrome | 2006 | 2009 | Observational | Belgium AND Spain | TTTS | Fetoscopic-laser |
| 41 | Maschke-2009 | Growth after intrauterine laser coagulation for twin–twin transfusion syndrome |  |  | observational |  | TTTS | Fetoscopic-laser |
| 42 | Matsushima-2014 | Neurodevelopmental impairment at three years of age after fetoscopic laser surgery for twin-to-twin transfusion syndrome | 2003 | 2014 | Observational |  | TTTS | Fetoscopic-laser |
| 43 | McIntosh-2014 | Long term developmental outcomes of pre-school age children following laser surgery for twin-to-twin transfusion syndrome | 2006 | 2008 | observational |  | ttts | Fetoscopic-laser |
| 44 | Mesbah-2022 | Infantile neurodevelopmental outcome after fetoscopic laser photocoagulation for twin-to-twin transfusion syndrome: the first prospective experience from Iran | 2018 | 2021 | observational |  | TTTS | Fetoscopic-laser |
| 45 | Middeldorp(1)−2007 | Fetoscopic Laser Surgery in 100 Pregnancies with Severe Twin-to-Twin Transfusion Syndrome in the Netherlands | 2000 | 2004 | observational |  | TTTS | Fetoscopic-laser |
| 46 | Middeldorp(2)-2007 | Twin-to-twin transfusion syndrome after 26 weeks of gestation: is there a role for fetoscopic laser surgery? | 1991 | 2006 | observational |  | TTTS | Fetoscopic-laser&Amnioreduction |
| 47 | Mieghem-2013 | Neurodevelopmental impairment at three years of age after fetoscopic laser surgery for twin-to-twin transfusion syndrome | 2003 | 2014 | Observational |  | TTTS | Fetoscopic-laser |
| 48 | Moise-2005 | A randomized trial of amnioreduction versus septostomy in the treatment of twin-twin transfusion syndrome | 1997 | 2002 | RCT |  | TTTS | Amnioreduction&septostomy |
| 49 | Mullers-2015 | Outcome following selective fetoscopic laser ablation for twin to twin transfusion syndrome: an 8 year national collaborative experience | 2006 | 2014 | observational |  | TTTS | Fetoscopic-laser |
| 50 | Peraltaa-2013 | Endoscopic Laser Dichorionization of the Placenta in the Treatment of Severe Twin-Twin Transfusion Syndrome | 2008 | 2012 | observational | spain | TTTS | Fetoscopic-laser |
| 51 | Persico-2015 | Postnatal survival after endoscopic equatorial laser for the treatment of twin-to-twin transfusion syndrome | 2011 | 2014 | observational | Italy | TTTS | Fetoscopic-laser |
| 52 | Pruetz-2011 | Twin–twin transfusion syndrome treated with laser surgery: postnatal prevalence of congenital heart disease in surviving recipients and donors | 2009 | 2010 | observational |  | TTTS | Fetoscopic-laser |
| 53 | Quintero-2003 | Stage-based treatment of twin-twin transfusion syndrome | 2003 |  | observational |  | TTTS | Fetoscopic-laser&Amnioreduction |
| 54 | Qunitero-2000 | selective versus non-selective laser photocoagulation of placental vessles in TTTS | 1994 | 1999 | observational |  | TTTS | Fetoscopic-laser |
| 55 | Ruegg-2018 | Outcome after fetoscopic laser coagulation in twin–twin transfusion syndrome – is the survival rate of at least one child at 6 months of age dependent on preoperative cervical length and preterm prelabour rupture of fetal membranes? | 2008 | 2014 | Observational | swiss | TTTS | Fetoscopic-laser |
| 56 | Rusticoa-2012 | Fetal and Maternal Complications after Selective Fetoscopic Laser Surgery for Twin-to-Twin Transfusion Syndrome: A Single-Center Experience | 2004 | 2009 | observational | Italy | TTTS | Fetoscopic-laser |
| 57 | Sago-2010 | The outcome and prognostic factors of twin–twin transfusion syndrome following fetoscopic laser surgery | 2002 | 2006 | observational |  | TTTS | Fetoscopic-laser |
| 58 | Salomon-2010 | Long-term developmental follow-up of infants who participated in a randomized clinical trial of amniocentesis vs laser photocoagulation for the treatment of twin-to-twin transfusion syndrome | 1999 | 2002 | observational |  | TTTS | Fetoscopic-laser |
| 59 | Senat-2004 | Endoscopic Laser Surgery versus Serial Amnioreduction for Severe Twin-to-Twin Transfusion Syndrome |  |  | observational |  | TTTS | Fetoscopic-laser&Amnioreduction |
| 60 | Sepulveda-2007 | Endoscopic laser surgery in severe second-trimester twin-twin transfusion syndrome: a three-year experience from a Latin American center | 2003 | 2006 | observational |  | TTTS | Fetoscopic-laser |
| 61 | Seshadri-2020 | NTRAFETAL LASER FOR MIDTRIMESTER TRAP SEQUENCE–EXPERIENCE FROM A SINGLE CENTER | 2011 | 2015 | observational | india | TTTS | Fetoscopic-laser |
| 62 | Shinar-2021 | Selective fetal reduction in complicated monochorionic twin pregnancies: a comparison of techniques | 1999 | 2019 | observational |  | TTTS | Fetoscopic-laser |
| 63 | Slaghekke(2)-2014 | Fetoscopic laser coagulation of the vascular equator versus selective coagulation for twin-to-twin transfusion syndrome: an open-label randomised controlled trial | 2008 | 2012 | RCT | five European | TTTS | Fetoscopic-laser |
| 64 | Snowise-2015 | Donor Death After Selective Fetoscopic Laser Surgery for Twin–Twin Transfusion Syndrome | 2011 | 2014 | observational | usa | TTTS | Fetoscopic-laser |
| 65 | Spruijt-2012 | Cerebral Injury in Twin–Twin Transfusion Syndrome Treated With Fetoscopic Laser Surgery | 2004 | 2011 | observational |  | TTTS | Fetoscopic-laser |
| 66 | Stirnemann-2021 | Intrauterine fetoscopic laser surgery versus expectant management in stage 1 twin-to-twin transfusion syndrome: an international randomized trial | 2011 | 2018 | RCT |  | TTTS | Fetoscopic-laser&Expectantmanagemnt |
| 67 | Swiatkowska-2012 | Results of laser therapy in twin-to-twin transfusion syndrome: our experience | 2005 | 2010 | observational | poland | TTTS | Fetoscopic-laser |
| 68 | Thia-2017 | Fetoscopic laser photocoagulation in twin-to-twin transfusion syndrome: experience from a single institution | 2011 | 2014 | observational |  | TTTS | Fetoscopic-laser |
| 69 | Tosello-2014 | Short and medium-term outcomes of live-born twins after fetoscopic laser therapy for twin-twin transfusion syndrome | 2007 | 2012 | observational |  | TTTS | Fetoscopic-laser |
| 70 | Valskya-2011 | Fetoscopic Laser Surgery for Twin-to-Twin Transfusion Syndrome after 26 Weeks of Gestation | 2006 | 2009 | observational |  | TTTS | Fetoscopic-laser |
| 71 | Vanderbilt-2012 | Prevalence and risk factors of cerebral lesions in neonates after laser surgery for twin-twin transfusion syndrome | 2006 | 2011 | observational | USA | TTTS | Fetoscopic-laser |
| 72 | Verbeek-2017 | Renal function in neonates with twin-twin transfusion syndrome treated with or without fetoscopic laser surgery | 2009 | 2016 | observational | Netherlands | TTTS | Fetoscopic-laser |
| 73 | Wagner-2009 | Short- and long-term outcome in stage 1 twin-to-twin transfusion syndrome treated with laser surgery compared with conservative management | 2000 | 2007 | observational | Netherlands | TTTS | Fetoscopic-laser |
| 74 | Yamamoto-2005 | Incidence and impact of perioperative complications in 175 fetoscopy-guided laser coagulations of chorionic plate anastomoses in fetofetal transfusion syndrome before 26 weeks of gestation | 1999 | 2004 | observational |  | TTTS | Fetoscopic-laser |
| 75 | Yang-2010 | Fetoscopic laser photocoagulation in the management of twin-twin transfusion syndrome: local experience from Hong Kong | 2002 | 2008 | observational | hong-kong | TTTS | Fetoscopic-laser |

| **Table 2: quality assessment** | | | |
| --- | --- | --- | --- |
| **row** | **Study (Author-year)** | **Study design** | **Quality assessment** |
| **Randomized trial** | |  | **Jaded score (max5)** |
|  | Crombleholme-2007 | Randomized controlled trial | 3 |
|  | Moise-2005 | Randomized controlled trial | 3 |
|  | Salomon-2010 | Randomized controlled trial | 3 |
|  | Slaghekke (2)-2014 | Randomized controlled trial | 4 |
|  | Stirnemann-2021 | Randomized controlled trial | 3 |
|  | Senat-2004 | Randomized controlled trial | 3 |
| **Case control study** | | **Study design** | **Newcastle Ottawa scale*(max9)** |
|  | Johnson-2001 | Case control study | 6 |
|  | Quintero-2003 | Case control study | 5 |
|  | Lopriore-2005 | Case control study | 7 |
|  | Gray-2006 | Case control study | 6 |
|  | Lopriore-2006 | Case control study | 6 |
|  | Lopriore-2007(2) | Case control study | 5 |
|  | Kowitt-2012 | Case control study | 6 |
|  | Spruijt-2012 | Case control study | 7 |
|  | Kempen-2016 | Case control study | 7 |
|  | Verbeek-2017 | Case control study | 6 |
|  | Bartin-2022 | Case control study | 7 |
| **Observational studies** | | **Study design** | **Newcastle Ottawa scale**(max10)** |
|  | Huber-2006 | Observational study | 7 |
|  | Anh-2022 | Observational study | 7 |
|  | Chmait-2015(2) | Observational study | 9 |
|  | Diehl-2017 | Observational study | 8 |
|  | Gordon-2017 | Observational study | 8 |
|  | Gordon-2022 | Observational study | 5 |
|  | Habli-2009 | Observational study | 8 |
|  | Has-2014 | Observational study | 7 |
|  | Herberg-2005 | Observational study | 6 |
|  | Ierullo-2007 | Observational study | 6 |
|  | Klink-2014 | Observational study | 8 |
|  | Knijnenburg-2021 | Observational study | 8 |
|  | Korsakissok-2018 | Observational study | 7 |
|  | Loh-2020 | Observational study | 7 |
|  | Lombardo-2011 | Observational study | 4 |
|  | Lopriore-2007(1) | Observational study | 7 |
|  | Mari-2000 | Observational study | 4 |
|  | Matsushima-2014 | Observational study | 7 |
|  | McIntosh-2014 | Observational study | 6 |
|  | Mesbah-2022 | Observational study | 6 |
|  | Middeldorp(2)-2007 | Observational study | 5 |
|  | Middeldorp(1)−2007 | Observational study | 4 |
|  | Mullers-2015 | Observational study | 5 |
|  | Persico-2015 | Observational study | 7 |
|  | Pruetz-2011 | Observational study | 8 |
|  | Sago-2010 | Observational study | 6 |
|  | Snowise-2015 | Observational study | 7 |
|  | Swiatkowska-2012 | Observational study | 5 |
|  | Vanderbilt-2012 | Observational study | 6 |
|  | Yamamoto-2005 | Observational study | 6 |
|  | Hecher-2000 | Observational study | 7 |
|  | Qunitero-2000 | Observational study | 7 |
|  | Sepulveda-2007 | Observational study | 6 |
|  | Maschke-2009 | Observational study | 4 |
|  | Wagner-2009 | Observational study | 7 |
|  | Chmait-2010 | Observational study | 8 |
|  | Yang-2010 | Observational study | 7 |
|  | Chmait-2011 | Observational study | 6 |
|  | Gray-2011 | Observational study | 6 |
|  | Martinez-2011 | Observational study | 5 |
|  | Valskya-2011 | Observational study | 6 |
|  | Chang-2012(1) | Observational study | 6 |
|  | Chang-2012(2) | Observational study | 5 |
|  | Rusticoa-2012 | Observational study | 7 |
|  | Mieghem-2013 | Observational study | 7 |
|  | Peraltaa-2013 | Observational study | 7 |
|  | Tosello-2014 | Observational study | 8 |
|  | Klink-2015 | Observational study | 6 |
|  | Chmait-2017 | Observational study | 7 |
|  | Thia-2017 | Observational study | 6 |
|  | Ruegg-2018 | Observational study | 7 |
|  | Fang-2019 | Observational study | 7 |
|  | Bergh-2020 | Observational study | 8 |
|  | Anh-2021 | Observational study | 7 |
|  | Bamberg-2021 | Observational study | 9 |
|  | Duy-Anh-2022 | Observational study | 5 |
|  | Seshadri-2020 | Observational study | 6 |
|  | Shinar-2021 | Observational study | 6 |

**Supplementary 3: reported complications for each treatment modality**

The most common reported complication in the studies were PROM, repeated TTTS, and TAPS. PROM was the most reported complication after Fetoscopic laser surgery (23%). 1795 cases of 7042 pregnancy cases reported PROM after laser therapy (34 studies) (fig.). Repeated TTTS after laser occurred in 156 cases of 3632 pregnancy cases (3%) (18 studies). TAPS is reported in 255 cases of 3241 pregnancy (6%) (15 studies). Bleeding after laser occurred in (16 studies) 79 cases of 2472 pregnancy cases. Rate of reported cerebral palsy based on 17 studies that used laser surgery was 2 percentage (126 cases of 3983 cases). While the rate in studies used amnioreduction was 4 percentage (11 of 222 patients) (see figures 1-6).


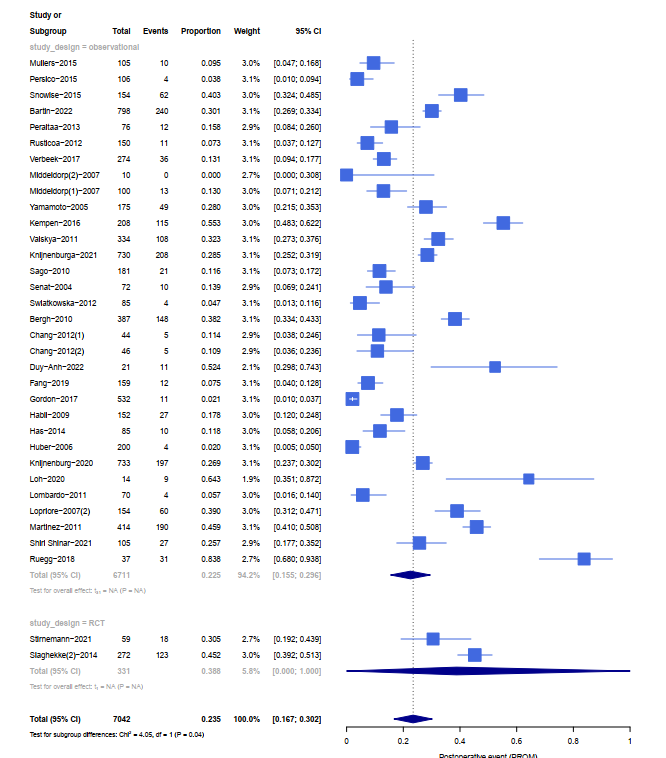


Figure 1. Forest plot showing PROM complication subdivided by type of studies of Fetoscopic laser surgery for TTTS


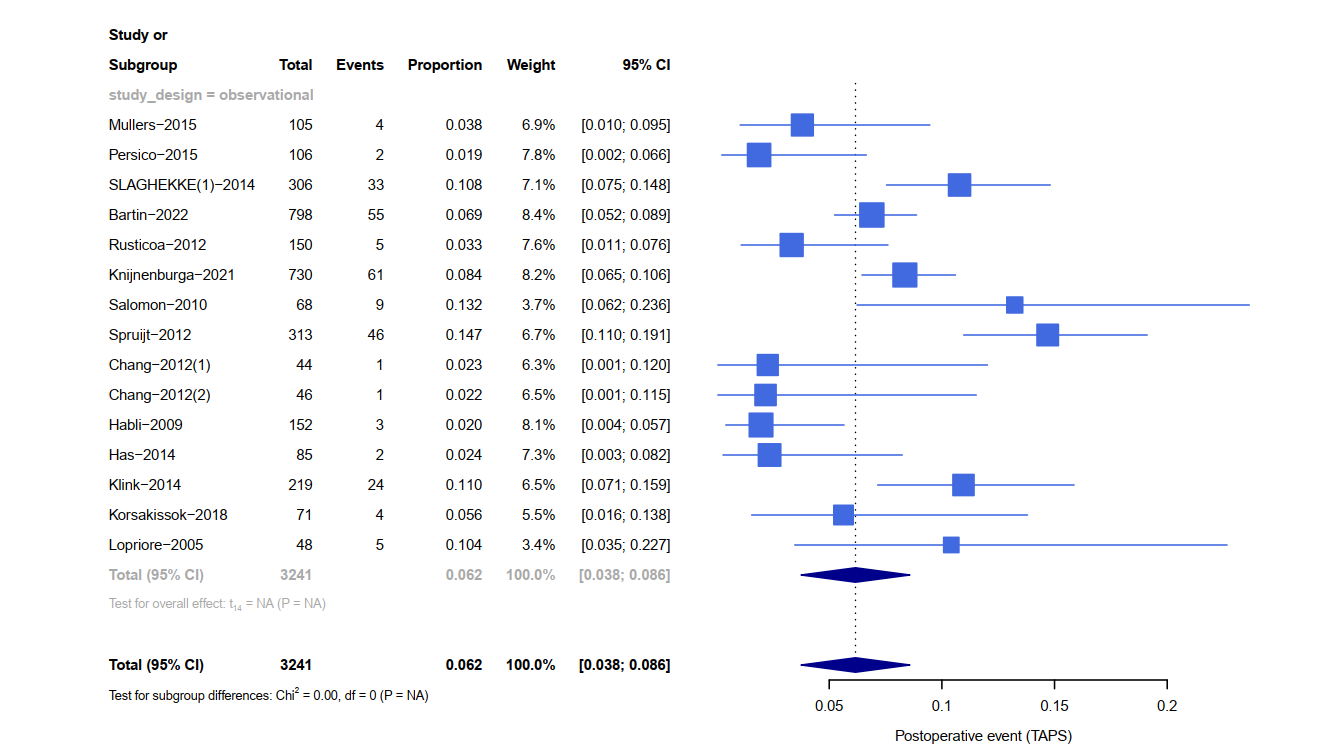


Figure 2. Forest plot showing TAPS complication subdivided by type of studies of Fetoscopic laser surgery for TTTS


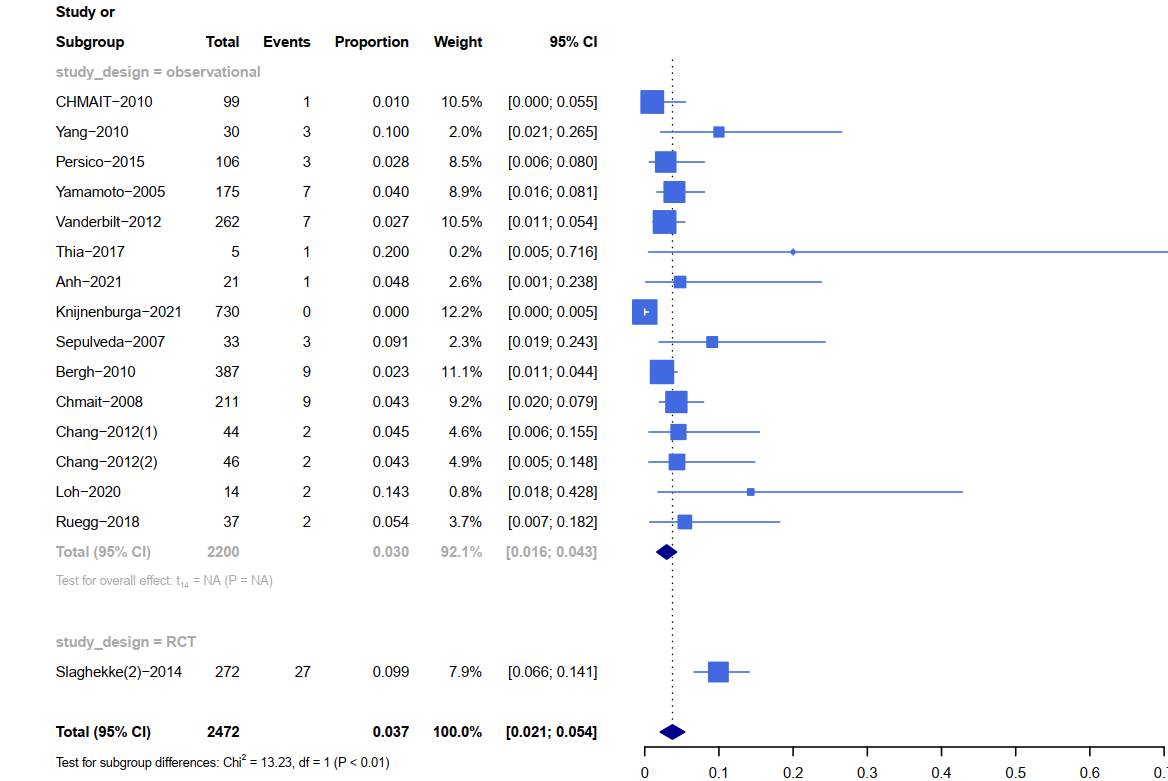


Figure 3. Forest plot showing bleeding complication subdivided by type of studies of Fetoscopic laser surgery for TTTS


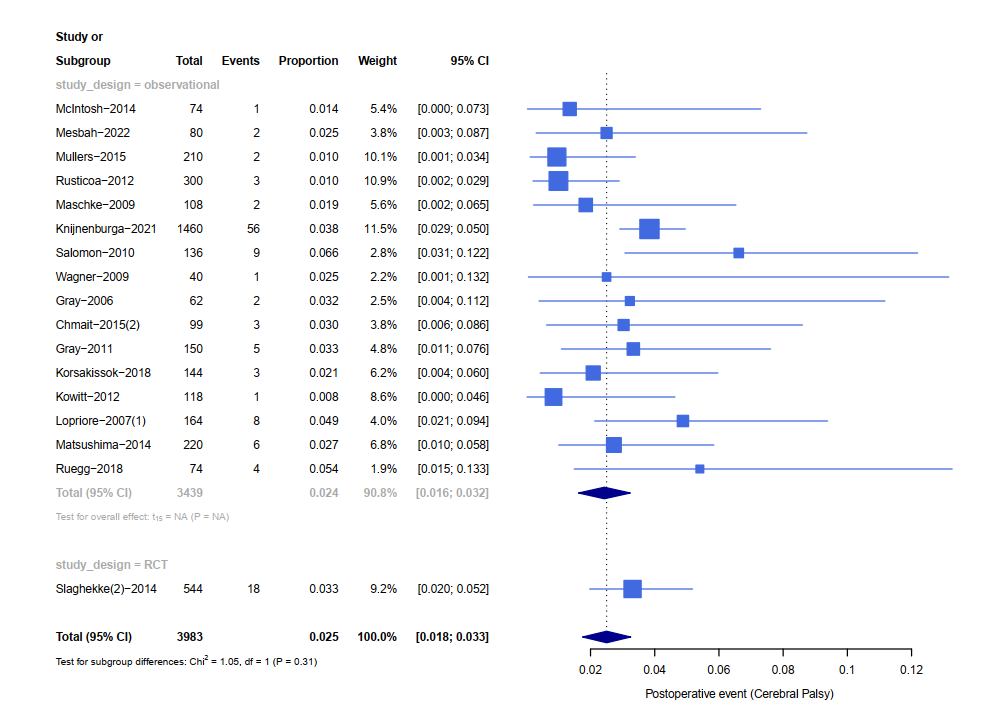


Figure 4. Forest plot showing cerebral lesion complication subdivided by type of studies of Fetoscopic laser surgery for TTTS


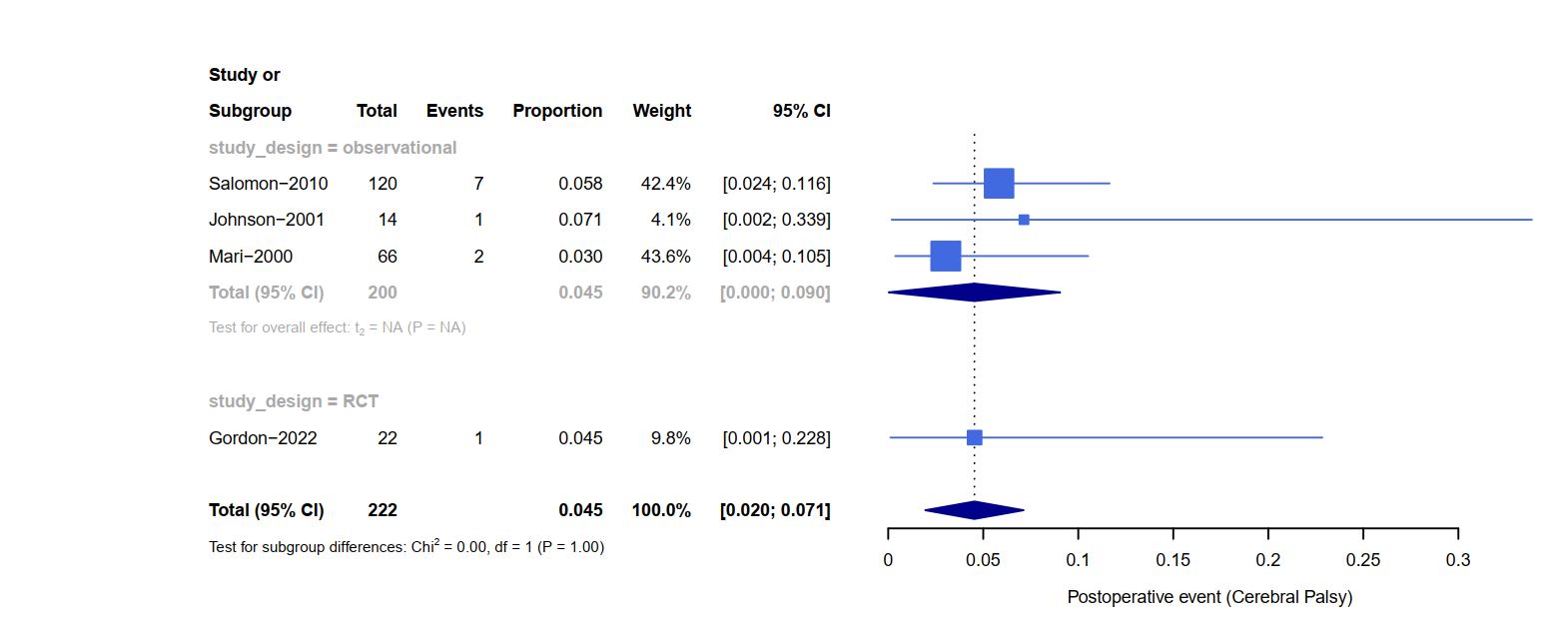


Figure5. Forest plot showing cerebral lesion complication subdivided by type of studies of Amnioreduction surgery for TTTS


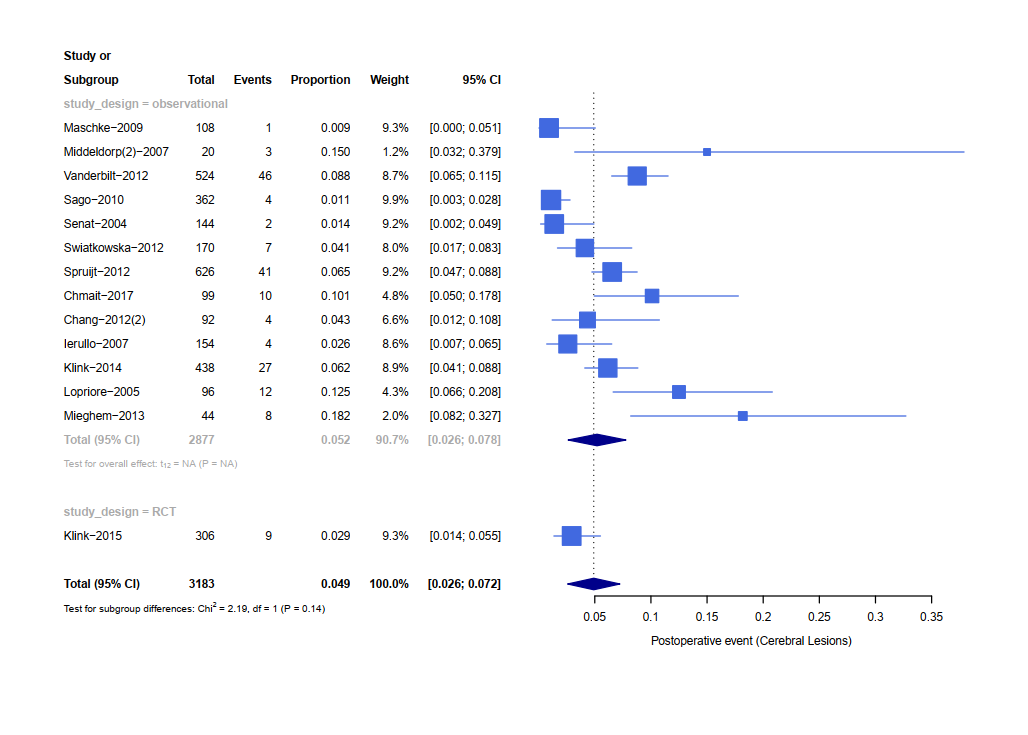


Figure 6. Forest plot showing cerebral palsy complication subdivided by type of studies of Fetoscopic laser surgery for TTTS
